# Omnivorous and plant-based dietary patterns: a comparative analysis using data-driven and index-based approaches

**DOI:** 10.1007/s00394-026-04046-z

**Published:** 2026-07-11

**Authors:** Eduardo Casas-Albertos, Noelia María Rodríguez-Martín, Ángela Alcalá-Santiago, Beatriz Sarriá, Mireia Obón-Santacana, Belén García-Villanova, María Dolores Ruiz-López, Mar Requena-Mullor, Ana Jimenez-Zabala, Miguel Rodriguez-Barranco, María-José Sánchez, Adela Castelló-Pastor, Esther Molina-Montes

**Affiliations:** 1 Department of Nutrition and Food Science, Faculty of Pharmacy, University of Granada, Campus Universitario de Cartuja s/n., 18071 Granada, Spain; 2 Biomedical Research Centre, Institute of Nutrition and Food Technology (INYTA) ‘José Mataix’, University of Granada, Avenida del Conocimiento s/n, 18071 Granada, Spain; 3 Instituto de Investigación Biosanitaria Ibs.GRANADA, Granada, Spain; 4 Group of Plant Protein, Department of Food and Health, Instituto de la Grasa-CSIC, Campus Universitario Pablo de Olavide, Edificio 46, Carretera de Utrera Km. 1, 41013 Seville, Spain; 5 Department of Metabolism and Nutrition, Institute of Food Science, Technology and Nutrition (ICTAN-CSIC), Spanish National Research Council (CSIC), José Antonio Nováis 10, 28040 Madrid, Spain; 6 Department of Nutrition and Food Science, Faculty of Pharmacy, University Complutense of Madrid, 28040 Madrid, Spain; 7 Oncology Data Analytics Program (ODAP), Unit of Biomarkers and Susceptibility (UBS), Catalan Institute of Oncology (ICO), L’Hospitalet del Llobregat, 08908 Barcelona, Spain; 8 ONCOBELL Program, Bellvitge Biomedical Research Institute (IDIBELL), L’Hospitalet de Llobregat, 08908 Barcelona, Spain; 9 Consortium for Biomedical Research in Epidemiology and Public Health (CIBERESP), 28029 Madrid, Spain; 10 Department of Nursing, Physical Therapy and Medicine, University of Almeria, 04120 Almeria, Spain; 11 Ministry of Health of the Basque Government, Sub Directorate for Public Health and Addictions of Gipuzkoa, San Sebastian, Spain; 12 Bioguipuzkoa Health Research Institute, Epidemiology of Chronic and Communicable Diseases Group, San Sebastián, Spain; 13 Andalusian School of Public Health (EASP), 18071 Granada, Spain; 14 Department of Chronic Diseases, National Centre for Epidemiology, Carlos III Institute of Health, Calle de Melchor Fernández Almagro, 5, 28029 Madrid, Spain

**Keywords:** Vegetarian, Vegan, Omnivorous, Diet index, Principal component analysis, Clustering

## Abstract

**Background and objective:**

Plant-based diet (PBD) followers are growing worldwide. The aim was to explore the dietary profile of PBDs, taking the omnivorous (OMN) diet as a reference.

**Methods:**

A cross-sectional study was carried out. A total of 760 participants were included, of whom 161 self-identified as vegan, ovo-lacto-vegetarian, or pesco-vegetarian. Intakes of 175 foods, assessed through a food frequency questionnaire (FFQ), were adjusted for energy intake. The intake of 32 food groups was compared across diet types using Kruskal–Wallis and post-hoc tests. Adherence to established a priori dietary patterns (Pro-vegetarian, Mediterranean, and EAT-Lancet) diet was assessed. A posteriori dietary patterns were characterized using principal component analysis (PCA), k-means and hierarchical clustering, amongst others.

**Results:**

Per 1000 kcal, daily mean intakes of legumes (> 48.2 g), vegetables/mushrooms (> 209.9 g), nuts (> 10.2 g), and whole grains (> 24.7 g) were higher in the PBD groups than OMN (*p* < 0.001). The three a priori dietary indices were inter-correlated (rho = 0.4–0.6) and OMN scored lower than the PBD groups (*p* < 0.001). PCA revealed a PBD-like pattern (variance: 15.0%), an unhealthy (7.2%), and a mixed animal-PBD pattern (5.5%). The clustering methods identified similar groupings of the PBD participants, and a group of PBD-like OMN (N = 61). The latter, compared with the other OMN patterns, showed significantly lower intakes of milk (48.0 vs. > 95.2 ml), red meat (13.9 vs. > 17.8 g), processed meat (9.0 vs. > 14.0 g), and precooked foods (9.0 vs. > 12.2 g), alongside higher intakes of whole grains (25.5 vs. < 20.8 g), nuts (13.8 vs. < 8.3 g), and vegetables (207.7 vs. < 161.3 g).

**Conclusions:**

Thus, food choices between OMN, vegetarians and vegans differ, beyond the exclusion of animal foods. PBDs present common characteristics despite their diversity, and some OMN share these dietary features.

**Graphical abstract:**

**Figure:**
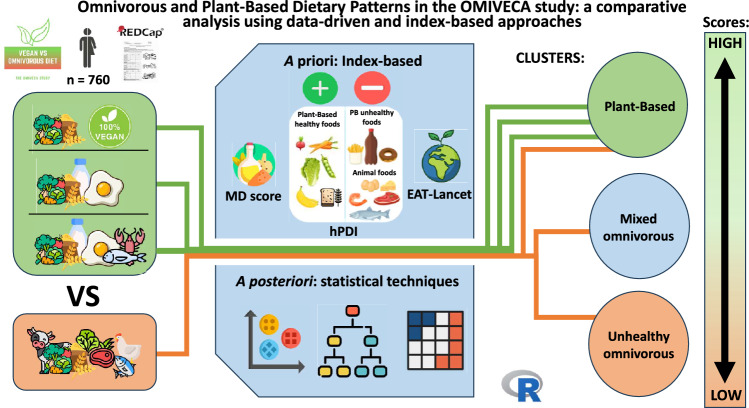

**Supplementary Information:**

The online version contains supplementary material available at 10.1007/s00394-026-04046-z.

## Introduction

In recent years, there has been a notable increase in the number of people adopting dietary patterns with reduced meat consumption, although with a varying extent [1]. Indeed, the prevalence of veganism and vegetarianism reaches 6% in some European countries and rises to 39% if flexitarians are considered [2]. This data is further supported by the increasing sales of vegetarian and plant-based (PB) alternative foods, with projections indicating a continued growth in the coming years [3, 4].

There are three main drivers behind this trend. First, plant-based diets (PBDs) are perceived as healthier alternatives [5, 6], due to their potential preventive effects against ischemic heart disease, diabetes, and cancer [7]. This has been attributed to their high content of fiber, and of bioactive compounds [8, 9], as well as the lower content of saturated fatty acids [10]. Second, the environmental impact of PBDs is significantly lower than that of animal-based diets, particularly in terms of carbon footprint and natural resource use [11, 12]. Third, ethical concerns regarding animal welfare also play a significant role in the adoption of PBDs [13].

However, the health effects of strict PBDs diets is a subject of ongoing debate given that the intake of vitamin B12, vitamin D and calcium, heme-iron, iodine, and zinc often fall below recommended levels [14–17]. Furthermore, the growth of PB alternatives as substitutes for animal-based products might not present the expected health benefits as many of these products are classified as ultra-processed foods despite their well-balanced nutritional profile. Also, while PBDs are often treated as a homogeneous group, they can encompass a wide spectrum of food choices ranging from flexitarian, pesco-, ovo- and lacto-vegetarian to VGN diets [18–20]. Similar to omnivorous diets, intergroup heterogeneity is also plausible as certain subgroups may adopt distinct dietary patterns; however, this issue remains unexplored.

Within this context, it has become essential to characterize PB dietary patterns. Several a priori diet indices have been developed for this purpose, including the Plant-based diet index (PDI) [21], which is an extension of the provegetarian food pattern developed in 2014 [22]; the Mediterranean diet (MD) Score [23], which prioritizes whole grains, olive oil, fish, fruits, nuts, and legumes, while penalizing meat, sugars and animal fat; and the EAT-Lancet Index [11], which promotes PB food intakes, reduced consumption of animal-based products, and incorporates both health and sustainability dimensions. All these indices assess the adherence to dietary patterns with high intake of PB foods, whereas the MD Score and EAT-Lancet Index account for a low consumption of animal products, considering different predefined levels of meat intake. In addition, a posteriori approaches (also known as “data-driven”) based on multivariate statistics have been used to cluster individuals according to their food consumption. Principal Component Analysis (PCA) is a classical approach in dietary pattern analysis [24]. However, other methods commonly used in multi-dimensional analyses of omics data could be applied to identify underlying data-driven dietary patterns. To date, few studies have explored them in the context of dietary pattern analysis [25, 26]. For instance, Partial Least Squares Discriminant Analysis (PLS-DA) which is a supervised method that minimizes the variables involved in the analysis to facilitate the clustering, as well as k-means and Hierarchical Clustering Analysis (HCA), which are non-supervised methods that are based on grouping the variables while exploring the relationship between them, represent new avenues in dietary pattern analysis [27, 28]. Recently, network food analysis has emerged as another approach for exploring dietary patterns [29]. To the best of our knowledge, such methods have not yet been applied to characterize PBDs. Exploring PB dietary patterns is important to inform dietary guidelines, shape public health strategies, and guide personalized nutrition recommendations for individuals adopting PBDs. The combination of a priori and a posteriori approaches aids in the characterization of complex and heterogeneous dietary patterns.

Therefore, the aim of this study was to comprehensively explore dietary patterns within PBDs in a specific study population of university students and the general population, using both dietary indices and advanced statistical methods, in order to gain insights into their characteristics, food profiles, and potential health implications.

## Methods

### Study design and study population

A cross-sectional study was carried out within the OMIVECA study (oMIcs profiles of pro-VEgetarian diets and Cancer). It started in 2023 and is currently ongoing. It’s a multicentric Spanish study mainly composed of young adults with the aim of exploring the differences between PBDs and conventional OMN diet, focused on dietary patterns, metabolomics and genomics, as well as the intake and status of (poly)phenols and other bioactive compounds [9].

The study population initially included 838 participants with complete dietary information. To minimize misreporting bias, participants above the 95th percentile of total energy consumed (> 4500 kcal/d) and below 5th percentile (< 1200 kcal/d) were excluded (N = 78). The remaining 760 participants constituted the study population. They were recruited from the Universities of Granada, Madrid, Seville and Almería in Spain. Among them, a total of 200 participants were recruited across all study centers, as well as from other Spanish provinces to validate and broaden the extrapolation of the results. This population subgroup was composed of older participants, not university students nor related to nutrition field. Participants were recruited through university stands, lectures at student events, and snowball sampling. The latter was also used to recruit participants from the other population group. All agreed to participate voluntarily and provided informed consent. The OMIVECA study was conducted according to the guidelines of the Declaration of Helsinki, and it has been approved by the Ethics Committees of Granada (Acta 11/21 CEIM/CEI Provincial Granada) for the Autonomous Community of Andalusia, the Institute of Health Carlos III ISCIII (CEI PI 32_2023) and the Spanish National Research Council CSIC (2014/2023).

### Dietary assessment

Dietary data was collected from April 2023 until May 2025 using an online administered semi-validated, Food Frequency Questionnaire (FFQ) implemented through REDCap software [30] hosted at the ISCIII. This questionnaire was based on a validated FFQ of 137 food items [31], which was further adapted for the current study with additional items (Table S1). Moreover, a new section comprising 31 vegetarian and VGN foods were added in order to improve the assessment of dietary intake in both VGN/vegetarian and non-vegetarian populations (Table S2): PB beverages, vegetarian/VGN foods such as meat alternatives, precooked meals, spreads, pastries and dairies), based on a previous market analysis of these kind of foods [19], along with and expert consensus involving nutrition professionals and a representative group of vegetarians.

In addition, participants self-reported their dietary pattern though the question: What type of diet do you identify with?; the response options being: omnivorous (OMN) all types of food, VGN (exclusively plant-based foods), ovo-lacto-vegetarian (OVL) includes eggs and dairy in addition to PB foods, and pesco-vegetarian (PCV) diet, includes fish, eggs, and dairy products in addition to PB foods. Moreover, the dietary pattern was confirmed through the reported intakes collected through the FFQ.

The FFQ was fully completed by all the participants. Consumption frequency was reported using nine response options, ranging from “Never or less than once per month” to “6 or more times per day”. Then, frequencies were converted into grams per day consumption for each food item. For seasonal foods, consumption frequency was corrected by multiplying the number of months per year when the foods are available (Table S1). This adjustment was applied to avoid overestimating annual consumption of foods that are only available or traditionally consumed during limited periods of the year.

Energy intake was derived through the Spanish Food Composition Database BEDCA [32]. The foods added from the USDA Food Composition Database [33] were: corn oil, seaweed, cereal meat substitute, legume meat substitute, soy meat substitute, almond drink, oats drink, VGN pastries, VGN biscuits, guacamole, VGN ice cream, hummus, kombucha, PB protein not soy-related, VGN yogurt-like not soy-related, VGN precooked fried meals, VGN lasagna, VGN cheese, smoothies, tempeh, PB spreads, PB cheese-like spreads.

The intake of food in grams per day was adjusted by energy intake (1000 kcal). In addition, food items were aggregated into 19 general food groups in order to better describe the food consumption: dairy, egg, meat, fish, vegetables, legumes, fruits, cereals, snacks and pastries, sugars, fats, beverages, alcohol, sauces, spices, precooked foods, prepared foods, vegetarian foods and VGN dairy-like products (Table S2).

### Dietary indices based on a priori methods

Three a priori Plant-based Diet Indices (PDI) were calculated. The overall PDI (oPDI) was based on 18 specific food groups, following the methodology described by Satija et al*.* Each food group was categorized as animal-based food, healthy or unhealthy PB food (Table S3) [21]. PB food groups (with no distinction between healthy or unhealthy) were divided into quintiles of intake and assigned a score ranging from 1 to 5, based on the frequency of consumption, from lowest to highest. Animal-based foods were scored reversely (5–1).

Two further indices were built. Regarding the healthy Plant-based Diet Index (hPDI), healthy PB foods were assigned positive scores, whereas unhealthy PB foods and animal-based foods were scored inversely. Conversely, for the unhealthy Plant-based Diet Index (uPDI), unhealthy PB food groups were scored as positive and healthy and animal-based foods were scored reversely (Table S3). PB precooked meals were considered as an additional component, resulting in 19 components for this index. The scoring procedure was similar to that described for the oPDI, using quintiles and scorings from 1 to 5.

The three indexes were expressed as continuous variables ranging from 18 to 90 points (up to 95 points for the uPDI) and were categorized to establish adherence levels: “low” (18–49 points), “medium” (50–64 points) and “high” (≥ 65 points). In addition, to analyze the adherence to a high diet index, categories “low” and “medium” were combined into a single group.

The relative MD Index used in this study has been previously described [34], and consists of eight components. Six components characteristic of the MD scored positively: fruits and nuts, vegetables (excluding potatoes), legumes, fish, cereals (including whole grain fiber) and olive oil. The two negative components were: meat and meat products, and dairy. Positive components scored 0 points for the lowest tertile and 2 points for the highest tertile of intake and reversely for negative components. The total score ranged from 0 to 16 points. Adherence categories were: low (≤ 5), medium (6–9), and high (≥ 10).

The EAT-Lancet Diet Index was built following EAT-Lancet Commission recommendations of 2019 for a sustainable diet [35]. This index comprises 14 components (Table S4): whole grains, tubers and starchy vegetables, vegetables, fruits, dairy foods, beef, lamb and pork; chicken and other poultry, eggs, fish, dry beans and lentils, peas, soy foods, peanuts or tree nuts, added fats and added sugars. For each component meeting the recommended intake, 1 point was assigned. The total score ranged from 0 to 14 points; adherence was categorized further into low (≤ 9), intermediate (10–11), and high (≥ 12).

### Assessment of other variables

Demographic and lifestyle information was collected using 21 items including sex, age, self-reported weight and height, quality and quantity of sleep, and three physical activity (PA) items: one accounting for the intensity of PA in a typical week (intense, moderate and low), other on the duration of the activity, and a third item for sitting hours as indicator of sedentary behavior. The use of supplements was assessed by two additional items: yes/no and the name of the supplement, including iron, vitamin B12, other vitamins of the group B, w-3, multivitamins and minerals. Anthropometric measurements were provided by 248 participants (32.6%) while completing the online questionnaire following standardized protocols, of whom 225 participants also provided information on PA by means of the short International Physical Activity Questionnaire (IPAQ) [36]. Both body measurements and detailed PA data were used to the internal validity of the use of self-reported measurements and the PA data.

Body mass index (BMI) was calculated for all participants as weight (kg)/height^2^ (m^2^). Participants were then categorized into BMI groups according to World Health Organization criteria: underweight (BMI < 18.5), normal weight (18.5 ≤ BMI < 25), overweight (25 ≤ BMI < 30), and obese (BMI ≥ 30). Although weight and height were self-reported, the concordance with physical measures collected in the subsample of 248 participants was high (Pearson rho = 0.98 for weight and 0.96 for height).

Based on the responses of the PA items, participants were classified into three categories: high PA (intense for at least 1 h/day or moderate for at least 2 h/day), medium PA (intense for 30 min or less/day, moderate for at least 30 min and light for more than 1 h/day) and low PA (moderate PA for less than 30 min/day and light PA for less than 1 h/day). As aforementioned, the short IPAQ was completed by 29.6% of the participants. Both the 2-items abbreviated and complete IPAQ questionnaires showed a good concordance (*kappa* squared = 0.53).

### Statistical analyses

Differences in demographic, lifestyle and dietary variables across the four diet groups (OMN, PCV, OVL, VGN) were explored with basic descriptive statistics and bivariate tests. For the age, continuous versions of the dietary indices and the intake of foods and food groups, differences between diet groups were evaluated by the Kruskal–Wallis test, as these variables did not follow a normal distribution (Shapiro Wilk test, *p* < 0.05). Pairwise post-hoc comparisons were performed using the Wilcoxon test and *p* values were adjusted for multiple comparisons using the Benjamini–Hochberg procedure [37]. Continuous variables were described by means and standard deviations (SD) as well as by medians and interquartile range (IQR).

A logistic regression model was built to explore the association between adherence to a high hPDI (dependent variable) and the type of diet (independent variable), adjusting for sex, age, participation center and profession, as standard adjustment variables. A second multivariate model additionally adjusted for BMI, total energy intake, and PA, due to their well-established relationship with diet-related behaviors. Multicollinearity between these variables was found to be acceptable. In both models, the OMN diet was used as the reference category. Model performance was further confirmed using the likelihood ratio test and the Hosmer–Lemeshow goodness-of-fit test. Effect modification by sex, center (Granada or Other), profession and age was tested by including an interaction term with diet type. Odds Ratio (OR) and 95% confidence intervals (CI) were derived from these models. Additionally, Spearman correlations between diet indices were used to explore relationships among these indices.

### Dietary pattern analysis based on a posteriori methods

Six types of a posteriori methods were considered and thirty-two food subgroups were selected (Table S5), expressed as grams per day of intake, energy adjusted per 1000 kcal and scaled to z-scores.

PCA was performed in order to minimize the data dimensionality after verifying sampling adequacy (Kaiser–Meyer–Olkin measure, KMO > 0.5). A varimax rotation was applied, and the number of components retained was determined based on Kaiser’s criterion (eigenvalues > 1). Factor loadings (FL) of at least ± 0.2 were used to select and interpret the dietary patterns. Afterwards, K-means clustering was performed to classify the individuals based on the extracted dimensions.

PLS-DA was also performed on the food groups using type of diet to train the model and to derive the dietary patterns based on the dietary predictor variables. Variable Importance in Projection (VIP) and FL for every food group were obtained. Scatter-plot graphs were obtained to visualize the first two dimensions.

A t-distributed Stochastic Neighbor Embedding (t-SNE) analysis was carried out to improve visualization of diet type diet across the reduced dimensions. Graphical visualization was compared to those obtained from PCA and PLS-DA graphics.

K-means clustering was also carried out on the intakes of the 32 food subgroups. The optimal number of clusters was explored using elbow and silhouette plots. A 2-dimensional graph was also built in order to visualize the three clustering solution.

In addition, HCA was applied to classify both the study population and the food subgroups into three clusters. Likewise, the number of clusters was chosen according to the Silhouette method. We selected Ward’s method for clustering, using Euclidean distances for rows (food groups) and for columns (participants), to enhance graphic interpretability [38]. A heat-map with dendrograms was obtained to facilitate the interpretation of the results. The retaining clusters were related to variables including center, age, sex and type of diet. Sociodemographic characteristics and food groups intake differences were explored across the three clusters.

Lastly, network analysis was applied to the food groups. First, Spearman correlation matrices were computed between the intakes of the food groups within each dietary group. These correlations were visualized as circular network graphs, where node size indicated the overall connectivity of each food group, and edge color and width represented the direction and magnitude of the pairwise correlations. In parallel, radial plots were constructed to display the scaled intakes of food groups.

To assess the robustness of the results, the following sensitivity analyses were carried out: (i) identification of dietary patterns using data derived from three 24 h recalls in a subset of 230 participants; (ii) restricting the analyses to university students of Human Nutrition, for whom dietary data quality is expected to be higher; and iii) exclusion of male participants to assess the stability of the clusters among women. (iv) restricting the analyses to participants older than 25 years old.

A level of statistical significance of < 0.05 was considered in all analyses. Data analyses were conducted using R software version 4.4.2 [39], along with the following packages: dplyr [40], CompareGroups [41], ggplot2 [42], factoMineR [43], factoextra [44], pheatmap [45], mixOmics [46] and Rtsne [47].

## Results

### Characteristics of the study population

Table 1 shows the main characteristics of the study population. A total of 760 participants were included (mean age: 25.9 years). Among them, 560 participants (73.2% women) were university students in health-related fields, including Human Nutrition, of whom 418 were aged 18 to 25 years old, and 142 were older than 25 years. The group of VGN was the oldest (33.3 years), followed by the vegetarians (28.1 and 28.6 years). Significant differences were noted in BMI, with PCV presenting a lower BMI than OMN (*p* = 0.003) and OVL (*p* = 0.035) and smoking/alcohol habits. Most participants were recruited in Granada (74.2%) and the majority were Human Nutrition students (47.1%), with significant differences across the diet groups (*p* < 0.001). Supplement use was high among VGN (96.5%) and OVL (82.2%), whereas PCV and OMN were more frequent non-users (> 60%) (*p* < 0.001). Other variables, such as PA, snacking, and quality and quantity of sleep were not significantly different between groups.

**Table 1:** Characteristics of the OMIVECA study population

	All n = 760	OMN n = *599*	PCV n = 31	OVL n = 73	VGN n = 57	*p* value
Sex^a^						0.001
Male	204 (26.8%)	172 (28.7%)	1 (3.2%)	11 (15.1%)	20 (35.1%)	
Female	556 (73.2%)	427 (71.3%)	30 (96.8%)	62 (84.9%)	37 (64.9%)	
BMI^b^ (kg/m^2^)	22.3 (20.4;24.4)	22.4 (20.6;24.5)	20.2 (19.6;22.9)	22.0 (20.5;24.2)	21.7 (20.3;24.2)	0.007
Age^a^						< 0.001
26 or more	263 (34.6%)	167 (27.9%)	17 (54.8%)	38 (52.1%)	41 (71.9%)	
Less than 26	497 (65.4%)	432 (72.1%)	14 (45.2%)	35 (47.9%)	16 (28.1%)	
Center						NA
Granada	564 (74.2%)	473 (79.0%)	23 (74.2%)	45 (61.6%)	23 (40.4%)	
Madrid	68 (8.9%)	44 (7.3%)	2 (6.5%)	12 (16.4%)	10 (17.5%)	
Seville	43 (5.7%)	26 (4.3%)	1 (3.2%)	7 (9.6%)	9 (15.8%)	
Almería	55 (7.2%)	48 (8.0%)	4 (12.9%)	3 (4.1%)	0 (0.0%)	
Other	30 (3.9%)	8 (1.3%)	1 (3.2%)	6 (8.2%)	15 (26.3%)	
Alcohol^c^						0.011
Never	450 (59.2%)	366 (61.1%)	11 (35.5%)	35 (47.9%)	38 (66.7%)	
Once per week	236 (31.1%)	179 (29.9%)	17 (54.8%)	28 (38.4%)	12 (21.1%)	
≥ 2 per week	74 (9.7%)	54 (9.0%)	3 (9.7%)	10 (13.7%)	7 (12.3%)	
Smoking^c^						0.002
Never	628 (82.6%)	509 (85.0%)	22 (71.0%)	60 (82.2%)	37 (64.9%)	
Former smoker	77 (10.1%)	50 (8.3%)	7 (22.6%)	7 (9.6%)	13 (22.8%)	
Currently	55 (7.2%)	40 (6.7%)	2 (6.5%)	6 (8.2%)	7 (12.3%)	
Profession^a^						< 0.001
Nutrition/dietetics	358 (47.1%)	306 (51.1%)	10 (32.3%)	29 (39.7%)	13 (22.8%)	
Health sciences	202 (26.6%)	176 (29.4%)	11 (35.5%)	13 (17.8%)	2 (3.5%)	
Other	200 (26.3%)	117 (19.5%)	10 (32.3%)	31 (42.5%)	42 (73.7%)	
Supplements use^a^						< 0.001
Yes	272 (35.8%)	145 (24.2%)	12 (38.7%)	60 (82.2%)	55 (96.5%)	
No	488 (64.2%)	454 (75.8%)	19 (61.3%)	13 (17.8%)	2 (3.5%)	
Physical activity^a^						0.795
High	284 (37.4%)	223 (37.2%)	11 (35.5%)	26 (35.6%)	24 (42.1%)	
Moderate	379 (49.9%)	296 (49.4%)	18 (58.1%)	40 (54.8%)	25 (43.9%)	
Low	97 (12.8%)	80 (13.4%)	2 (6.5%)	7 (9.6%)	8 (14.0%)	
Sleep^a^ (good quality)	433 (57.0%)	339 (56.6%)	17 (54.8%)	48 (65.8%)	29 (50.9%)	0.354
Sleep hours^c^						0.537
< 6 h	89 (11.7%)	73 (12.2%)	3 (9.7%)	7 (9.6%)	6 (10.5%)	
6–8 h	606 (79.7%)	476 (79.5%)	27 (87.1%)	61 (83.6%)	42 (73.7%)	
> 8 h	65 (8.6%)	50 (8.3%)	1 (3.2%)	5 (6.8%)	9 (15.8%)	
Snacking^a^						0.542
Yes	294 (38.7%)	224 (37.4%)	13 (41.9%)	31 (42.5%)	26 (45.6%)	
No	466 (61.3%)	375 (62.6%)	18 (58.1%)	42 (57.5%)	31 (54.4%)	

*NA* not available; *OMN* omnivorous; *VGN* vegan; *OVL* ovo-lacto-vegetarian; *PCV* pesco-vegetarian

^a^ n (%) for categorical variables; *p*-value derived from Pearson Chi-square test

^b^ Median and interquartile range for continuous variables (BMI); *p* value derived from Kruskal–Wallis test

^c^ n (%) for categorical variables; *p*-value derived from Fisher’s exact test

### Food intake by diet groups

**Fig. 1:**
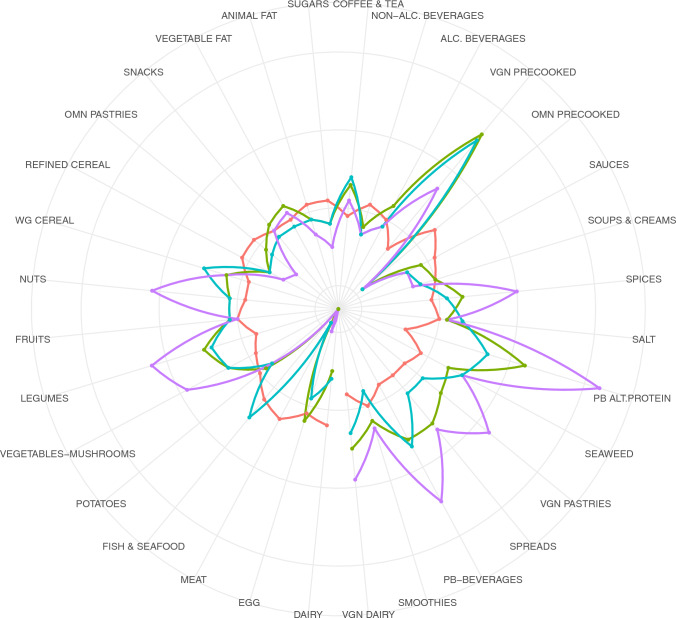
Radial graph of the main food groups dietary intake (scaled) by type of diet in the OMIVECA study. The graph displays the normalized (z-score) intakes of 32 food groups. Red represents the OMN diet, blue the PCV diet, green the OVL diet, and purple the VGN diet. Intakes and post-hoc analyses to visualize the difference between the four groups are presented in Supplemental Tables 6 and 7. *Abbreviations* OMN: omnivorous; PCV: pesco-vegetarian; OVL: ovo-lacto-vegetarian; VGN: vegan

Figure 1 shows the intake of key food groups (standardized to the mean and SD) by diet type. VGN exhibited distinctive dietary profiles with higher intake of nuts, legumes, PB beverages and other PB foods. Details of the dietary intake of 65 food groups (g/d per 1000 kcal) are shown in Table S6 pairwise comparisons by diet group in Table S7 and raw intakes in Table S8. The consumption of PB foods was higher among VGN and vegetarians, following a descendent gradient from more to less restriction of animal foods. As expected, VGN reported no intake of animal foods whereas, OMN consumed, on average per 1000 kcal, 142.0 g/d of dairy products, 17.7 g/d of eggs, 61.2 g/d of meat and meat products, and 34.4 g/d of fish/seafood (all *p* < 0.001). On the other hand, OVL and PCV differed significantly only in fish consumption (mean intake in PCV: 40.9 g/d) and in some vegetarian foods: PB alternative proteins (48.2 vs 33.9 g/d) and spreads (5.1 vs 3.3 g/d), respectively. Intake of vegetables/mushrooms was notably higher in VGN (266.1 g/d), while about 100 g lower in OMN (*p* < 0.001). It was also significantly lower in OMN compared with OVL and PCV groups (209.9 g/d and 2013.8 g/d, respectively; *p* < 0.001). OVL and PCV reported an intermediate legume intake (~ 50 g/d; *p* < 0.05) compared to OMN and VGN. In contrast, no significant differences were observed for potatoes and fruits consumption.

For other PB foods, we observed a markedly greater intake of nuts and whole grain cereals among VGN (19.2 g/d and 25.2 g/d, respectively), as compared to the OMN group (8.0 g/d and 19.0 g/d, respectively; *p* < 0.001 and *p* = 0.002). VGN also reported a lower intake of sugars (1.8 g/d) than OMN (4.6 g/d), OVL and PCV (3.3 g/d), but a higher consumption of coffee/tea than OMN (32.1 g/d vs 27.1 g/d), and significantly higher use of spices (4.2 g/d vs 2–3 g/d; *p* < 0.001). The intake of cereals (sum of whole grains and refined cereals), as well as the amount of consumed vegetable fats and beverages (sugared, sweetened and juices), was similar between the groups. However, the total consumption of non-alcoholic beverages, excluding PB beverages, among OMN was higher than that of VGN and OVL (19.1 g/d vs 9–10 g/d, respectively; *p* = 0.013). On the other hand, the intake of PB beverages reached 108.5 g/d among VGN (vs 13.5 g/d in OMN; *p* < 0.001), an amount comparable to the milk intake of OMN. In contrast, OVL and PCV consumed only half the amount of PB beverages and milk compared to the other groups (*p* < 0.001). Finally, consumption of precooked meals was lower among VGN than the other groups (7.1 g/d vs 12–14 g/d; *p* < 0.001), whereas OMN did not differ from OVL and PCV groups (*p* = 0.250).

Finally, consumption of vegetarian-like foods (e.g. meat alternatives, protein alternatives, and spreads), were almost negligible among OMN (< 3 g/d) but higher among VGN (21.6 g/d, 76.2 g/d and 5.2 g/d in VGN, and 16–19 g/d, 34–48 g/d and 3–5 g/d in OLV and PCV, respectively). Only the consumption of protein alternatives (*p* = 0.01) and that of spreads (*p* = 0.04) differed significantly between the PBD groups.

### Adherence to a priori dietary indices by diet groups

The distribution into levels of adherence to the dietary indices reflecting PBDs by type of diet is shown in Table 2. VGN presented a higher median score of the oPDI (63 points), with a descendent gradient observed from vegetarians to OMN (52 points). Likewise, adherence to the hPDI was higher in VGN (74.0 points) followed by OVL (67.0 points), PCV (64.0 points), and OMN (53.0 points). Moreover, adherence across study groups was significantly different (*p* < 0.001), except between the OVL and PCV groups (Figure S1). In multivariate adjusted models for sex, age, center and profession, as well as BMI, physical activity and energy intake (Table S9), VGN had 42.26 times higher odds of adherence to a high hPDI compared to OMN (95% CI 17.81–115.31). The odds of adherence to the hPDI were greater in OVL (8.02; 95% CI 4.62–14.09) and PCV (3.95; 95% CI 1.77–8.72). BMI was inversely associated to adherence to a high hPDI; however, the significance threshold was not reached. There was no effect modification by sex (*p* = 0.261), age (*p* = 0.849), center (*p* = 0.204), profession (*p* = 0.106), BMI (*p* = 0.151), physical activity (*p* = 0.553), and energy intake (*p* = 0.571) (data not shown).

**Table 2:** Plant-based dietary indices by type of diet in the OMIVECA study

	All n = 760	OMN n = 599	PCV n = 31	OVL n = 73	VGN n = 57	*p* value
oPDI^a^	54.0 (49.0;59.0)	52.0 (48.0;56.0)	56.0 (50.0;60.5)	59.0 (56.0;63.0)	63.0 (61.0;68.0)	< 0.001
hPDI^a^	56.0 (48.0;64.0)	53.0 (47.0;60.0)	64.0 (55.0;68.5)	67.0 (61.0;70.0)	74.0 (69.0;79.0)	< 0.001
hPDI categorized^b^						< 0.001
Low (< 50)	214 (28.2%)	206 (34.4%)	4 (12.9%)	4 (5.5%)	0 (0.0%)	
Intermediate (50–64)	361 (47.5%)	315 (52.6%)	13 (41.9%)	26 (35.6%)	7 (12.3%)	
High (≥ 65)	185 (24.3%)	78 (13.0%)	14 (45.2%)	43 (58.9%)	50 (87.7%)	
uPDI^a^	54.0 (48.0;60.0)	54.0 (49.0;60.0)	51.0 (43.0;56.5)	51.0 (47.0;59.0)	54.0 (49.0;59.0)	0.031
uPDI categorized^c^						0.014
Low (< 50)	225 (29.6%)	163 (27.2%)	15 (48.4%)	32 (43.8%)	15 (26.3%)	
Intermediate (51–64)	473 (62.2%)	384 (64.1%)	16 (51.6%)	37 (50.7%)	36 (63.2%)	
High (≥ 65)	62 (8.2%)	52 (8.7%)	0 (0.0%)	4 (5.5%)	6 (10.5%)	
MD Score^a^	8.0 (6.0;10.0)	8.0 (6.0;10.0)	11.0 (10.0;12.0)	10.0 (9.0;11.0)	11.0 (10.0;12.0)	< 0.001
MD Score categorized^c^						< 0.001
Low (≤ 5)	112 (14.7%)	111 (18.5%)	0 (0.0%)	1 (1.4%)	0 (0.0%)	
Intermediate (6–9)	367 (48.3%)	329 (54.9%)	5 (16.1%)	23 (31.5%)	10 (17.5%)	
High (≥ 10)	281 (37.0%)	159 (26.5%)	26 (83.9%)	49 (67.1%)	47 (82.5%)	
EAT- Lancet score^a^	10.0 (9.0;11.0)	9.0 (9.0;11.0)	11.0 (10.0;12.0)	11.0 (10.0;12.0)	11.0 (10.0;12.0)	< 0.001
EAT- Lancet score categorized^c^						< 0.001
Low (≤ 9)	323 (42.5%)	301 (50.3%)	5 (16.1%)	13 (17.8%)	4 (7.0%)	
Intermediate (10–11)	333 (43.8%)	249 (41.6%)	14 (45.2%)	41 (56.2%)	29 (50.9%)	
High (≥ 12)	104 (13.7%)	49 (8.2%)	12 (38.7%)	19 (26.0%)	24 (42.1%)	

The Overall Provegetarian Diet Index (oPDI) and the Healthy Provegetarian Diet Index (hPDI) ranged from 18 to 90 points. The Unhealthy Provegetarian Diet Index (uPDI) ranged from 19 to 95 points. The Mediterranean Diet (MD) Score ranged from 0 to 16 points, and the EAT-Lancet Score ranged from 0 to 14 points.

*OMN* omnivorous; *VGN* vegan; *OVL* ovo-lacto-vegetarian; *PCV* pesco-vegetarian

^a^ Median and interquartile range for continuous indices; *p* value derived from Kruskal–Wallis test

^b^ n (%) for categorical indices; *p* value derived from Pearson Chi-square test

^c^ n (%) for categorical indices; *p* value derived from Fisher’s exact test

The correlations between the scores were moderate (rho: 0.4–0.6). Therefore, similar trends were observed for the other two a priori dietary indices. OMN showed the lowest adherence to the MD score and EAT-Lancet score while the three PBD groups obtained similar scores.

### A posteriori dietary patterns by diet groups

**Fig. 2:**
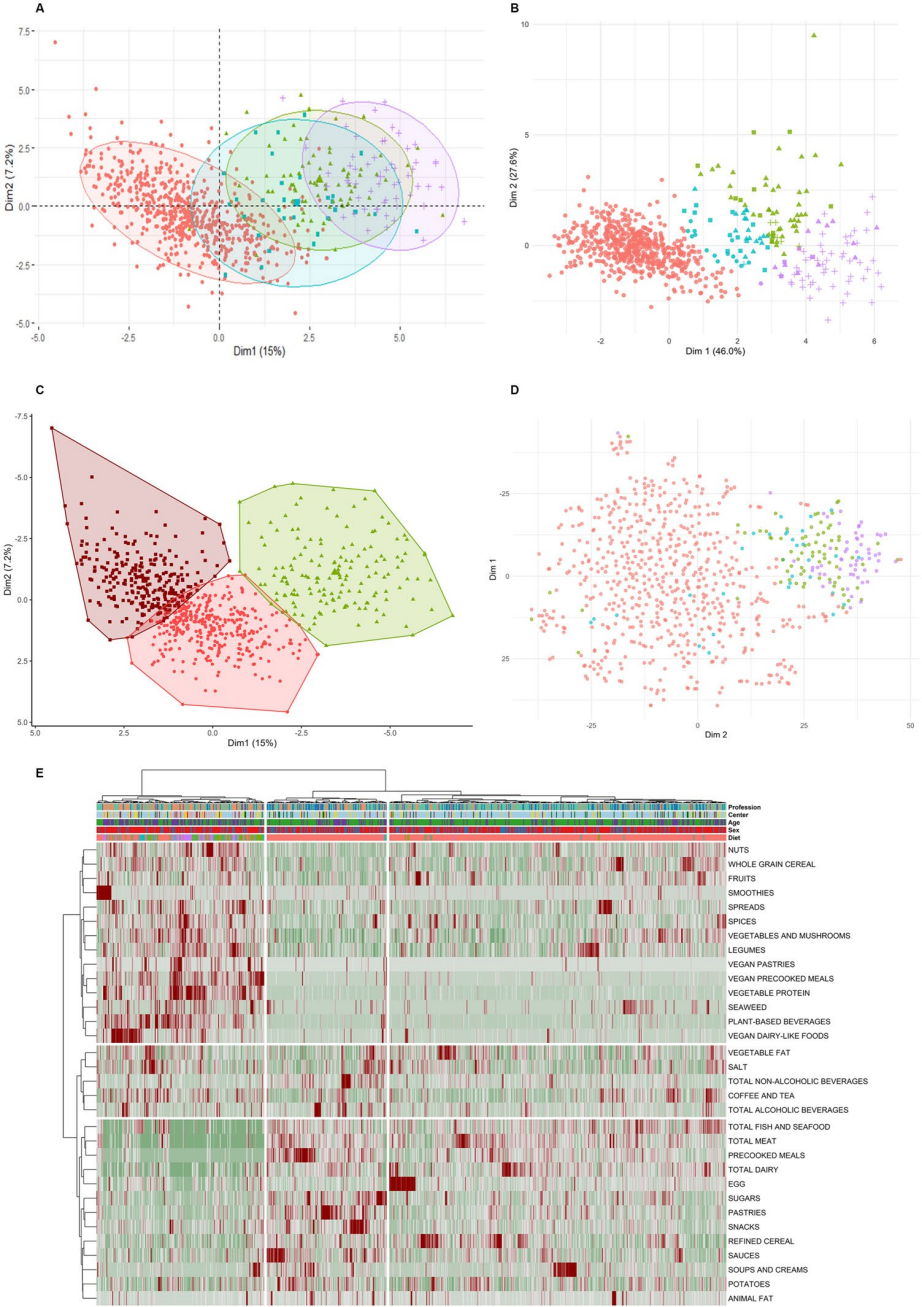
Dietary patterns and clusters derived in the OMIVECA study from different a posteriori methods: (A) PCA. (B) PLS-DA. (C) K-means. (D) t-SNE. (E) HCA heatmap. **A** PCA: Each dot represents an individual, colored by self-reported diet: red for OMN, green for OVL, blue for PVC, and purple for VGN. Ellipses represent the 90% confidence regions for each dietary group. Overlap indicates potential similarity or misclassification between groups. **B** PLS-DA: Each dot represents an individual, with shapes indicating the self-reported diet: circles for OMN, triangles for OVL, squares for PCV, and crosses for VGN. Colors reflect the predicted classification from the PLS-DA model: red for OMN, green for OVL, blue for PCV, and purple for VGN. **C** K-means: Individuals are grouped into three clusters: green represents a plant-based dietary pattern, dark red indicates OMN with an unhealthy profile, and light red corresponds to a mixed OMN diet. **D** t-SNE: Dots are colored by diet: red for OMN, green OVL, blue for PCV, and purple for VGN. **E** HCA: Red indicates higher intake and green lower intake. In the profession variable, light green indicates "Nutrition”, light blue indicates “Health Sciences students" and orange is for "Others". For center, blue is Granada, yellow Madrid, brown Seville, green Almería, and pink "Other". For age, green corresponds to individuals under 26 years, and blue to those aged 26 or older. In the sex variable, red represents females and blue males. Diet colors are consistent with the previous panels. *Abbreviations* PCA: principal component analysis; OMN: omnivorous; PCV: pesco-vegetarian; OVL: ovo-lacto-vegetarian; VGN: vegan; PLS-DA: partial-least squares discriminant analysis; t-SNE: t-distributed Stochastic Neighbor Embedding

The different dietary patterns derived from a posteriori methods are shown in Fig. 2. In PCA, three components were retained, explaining 27.7% of the total variance (Fig. 2A). FL for each component (> 0.2) are detailed in Table S10. The first component, explaining 15.0% of variance, was associated to a PBD. The strongest positive loadings (> 0.3) included legumes (FL = 0.426), PB beverages (FL = 0.555), PB precooked meals (FL = 0.644), PB protein (FL = 0.783), VGN pastries (FL = 0.440), PB spreads (FL = 0.409), and PB dairy-like foods (FL = 0.455). Negative loadings were animal-based foods, such as total meat (FL = − 0.676), fish/seafood (FL = − 0.556), and OMN precooked meals (FL = − 0.389).

The second PCA component (7.2% of explained variance), reflected an unhealthy OMN diet. Strong positive loadings were observed for pastries (FL = 0.550), snacks (FL = 0.592), sugars (FL = 0.468), non-alcoholic beverages (FL = 0.451), OMN precooked meals (FL = 0.496), and sauces (FL = 0.482). Negative loadings included vegetables/mushrooms (FL = − 0.484), legumes (FL = − 0.304), fruits (FL = − 0.467), nuts (FL = − 0.453), and whole grain cereals (FL = − 0.516).

The third component (5.5% of explained variance) represented an intermediate healthy OMN dietary pattern. The positive loadings included vegetable fat (FL = 0.553), coffee/tea (FL = 0.556), total alcoholic beverages (FL = 0.406), spices (FL = 0.304), salt (FL = 0.672), whereas negative loadings were primarily pastries (FL = − 0.255), and soups (FL = − 0.301).

After PCA dimensional reduction, three clusters were identified using the k-means approach (Table S11). The first cluster, representing intermediate mixed animal and PBD pattern, included 307 OMN (95.0%), and 16 (5.0%) PBD followers. This cluster featured a high consumption of dairy products (145.2 g/d), eggs (21.5 g/d), meat/processed meat (54.7 g/d), fish/seafood (36.5 g/d), vegetables/mushrooms (204.5 g/d), fruits (215.8 g/d), whole grain cereals (27.5 g/d), coffee/tea (33.9 g/d), and nuts (10.6 g/d).

The second cluster, characterized as a PBD pattern, comprised 10 OMN (6.5%), 57 VGN (37.3%), 67 OVL (43.8%), and 19 PCV (12.4%). Interestingly, 69.3% of the subjects in this cluster were classified as high hPDI adherence. In this cluster, food intake was characterized by low intakes of animal foods including dairy products (39.1 g/d), meat/processed meat (2.9 g/d), fish/seafood (7.1 g/d), as well as of unhealthy PB foods, such as refined cereals (23.7 g/d). In contrast, participants in this cluster reported a high consumption of vegetables/mushrooms (236.8 g/d), legumes (57.8 g/d), nuts (13.9 g/d), whole grain cereals (24.6 g/d), coffee/tea (35.4 g/d), VGN precooked meals (10.2 g/d), spices (3.4 g/d), PB protein (56.2 g/d), VGN pastries (2.5 g/d), PB spreads (5.1 g/d), smoothies (4.4 g/d), and VGN dairy-like products (20.9 g/d).

The third cluster, defined as an unhealthy dietary pattern, was composed almost entirely of OMN (282 participants; 99.3%), and very few OVL and PCV. Most participants (62.0%) in this cluster scored low in the hPDI. Compared to the previous cluster, the consumption of animal foods was significantly higher (dairy: 142.9 g/d, meat/processed meat: 66.0 g/d, and fish/seafood: 31.9 g/d), as was the intake of precooked meals (19.2 g/d), snacks (4.1 g/d), sugars (6.3 g/d), non-alcoholic beverages (28.8 g/d), sauces (7.7 g/d), and soups (6.1 g/d).

In PLS-DA, FLs for VIP > 1 and scatter plots agreed with the results obtained in PCA (Table S12, Fig. 2B). PBD followers were grouped in one main cluster, while a subset of OMN was classified as vegetarian-like. Consistent results were also obtained using k-means and t-SNE (Fig. 2C, D).

Similarly, HCA identified three main clusters (Fig. 2E). The food intake and other covariates of these clusters are shown in Table 3. The first cluster, aligned with PBDs, included 55 VGN (27.0%), 65 OVL (31.9%) and 23 PCV (11.3%), as well as a substantial proportion of OMN (29.9%). Participants in this cluster showed a high intake of vegetables/mushrooms (230.9 g/d), legumes (50.5 g/d), fruits (193.4 g/d), nuts (14.1 g/d), whole grain cereals (26.1 g/d), coffee/tea (37.8 g/d), spices (3.1 g/d), and VGN foods including vegetable proteins (44.2 g/d), PB beverages (77.3 g/d) and VGN dairy-like foods (19.6 g/d), but a low consumption of animal foods (meat/processed meat: 16.4 g/d, dairy products: 63.4 g/d, eggs: 12.7 g/d, and fish/seafood: 15.4 g/d).

**Table 3:** Dietary intake of main food groups and characteristics of the three clusters obtained by Hierarchical clustering

		Cluster 1: PBD n = 204	Cluster 2: Unhealthy OMN diet n = 146	Cluster 3: Mixed OMN diet n = 410	*p* value
Diet declared^a^				< 0.001	
	OMN	61 (29.9%)	142 (97.3%)	396 (96.6%)	
	VGN	55 (27.0%)	1 (0.7%)	1 (0.2%)	
	OVL	65 (31.9%)	1 (0.7%)	7 (1.7%)	
	PCV	23 (11.3%)	2 (1.4%)	6 (1.5%)	
Age (years)^a^	Less than 26	28.8 (9.8)	25.2 (10.1)	24.8 (8.5)	< 0.001
hPDI categorized^a^					< 0.001
	Low (< 50)	15 (7.4%)	86 (58.9%)	113 (27.6%)	
	Intermediate (51–64)	66 (32.4%)	57 (39.0%)	238 (58.0%)	
	High (≥ 65)	123 (60.3%)	3 (2.1%)	59 (14.4%)	
Sex^a^	Female	165 (80.9%)	94 (64.4%)	297 (72.4%)	0.003
BMI^c^ (kg/m^2^)		21.7 (20.3;23.9)	22.8 (20.3;25.0)	22.4 (20.6;24.4)	0.075
Food groups (g/d)					
Total dairy^d^		63.4 (73.9)	133.1 (78.0)	149.1 (95.2)	< 0.001
Egg^d^		12.7 (13.5)	12.2 (7.9)	19.9 (19.2)	< 0.001
Total meat^d^		16.4 (31.1)	62.1 (28.1)	59.7 (32.3)	< 0.001
Total fish & seafood^d^		15.4 (24.8)	31.8 (17.6)	34.5 (18.9)	< 0.001
Potatoes^d^		18.8 (15.4)	25.6 (17.4)	20.7 (14.3)	< 0.001
Vegetables & mushrooms^d^		230.9 (115.6)	120.3 (73.9)	173.4 (90.4)	< 0.001
Legumes^d^		50.5 (27.1)	26.7 (17.4)	35.6 (23.9)	< 0.001
Fruits^d^		193.4 (113.9)	136.7 (80.3)	192.0 (124.6)	< 0.001
Nuts^d^		14.1 (11.6)	4.8 (4.5)	8.3 (7.1)	< 0.001
Whole grain cereal^d^		26.1 (18.6)	10.9 (13.0)	21.0 (21.3)	< 0.001
Refined cereal^d^		22.7 (16.0)	35.1 (19.1)	35.7 (25.5)	< 0.001
Pastries^d^		6.1 (6.7)	20.8 (17.6)	8.7 (7.5)	< 0.001
Snacks^d^		2.4 (2.8)	5.2 (5.5)	1.8 (1.8)	< 0.001
Vegetable fat^d^		10.4 (6.3)	9.3 (6.4)	10.3 (6.4)	0.084
Animal fat^d^		0.3 (1.7)	0.5 (0.9)	0.4 (0.9)	< 0.001
Sugars^d^		2.6 (3.3)	7.7 (6.9)	3.8 (3.7)	< 0.001
Coffee & tea^d^		37.8 (32.6)	24.1 (25.3)	26.3 (26.7)	< 0.001
Total non-alc. beverages^d^		11.4 (18.4)	36.4 (55.5)	13.0 (17.2)	< 0.001
Total alc. beverages^d^		20.4 (34.7)	32.9 (56.6)	15.4 (21.4)	0.016
Vegan precooked meals^d^		8.0 (9.3)	0.9 (3.5)	0.8 (2.1)	< 0.001
Omnivorous precooked meals^d^		2.4 (5.2)	21.1 (15.6)	11.2 (8.5)	< 0.001
Sauces^d^		3.9 (3.9)	8.9 (7.1)	4.4 (3.9)	< 0.001
Soups & creams^d^		2.7 (6.7)	5.2 (7.2)	4.0 (7.7)	< 0.001
Spices^d^		3.1 (2.5)	1.6 (2.1)	1.9 (1.9)	< 0.001
Salt^d^		1.7 (1.3)	1.4 (1.1)	1.4 (1.0)	0.009
PB alt. protein^d^		44.2 (40.6)	2.5 (7.8)	3.0 (7.4)	< 0.001
Seaweed^d^		0.1 (0.3)	< 0.1 (0.1)	0.1 (0.1)	< 0.001
Vegan pastries^d^		1.8 (4.2)	0.3 (1.3)	0.1 (0.5)	< 0.001
Spreads^d^		4.4 (4.0)	2.0 (2.7)	2.3 (3.1)	< 0.001
PB beverages^d^		77.3 (80.3)	9.2 (29.0)	8.3 (21.3)	< 0.001
Smoothies^d^		5.8 (13.3)	1.9 (3.8)	1.5 (3.2)	< 0.001
Vegan dairy-like foods^d^		19.6 (26.3)	2.7 (9.1)	2.8 (7.8)	< 0.001

The food groups included were the 32 used to explore hierarchical clustering. The Healthy Provegetarian Diet Index (hPDI) ranged from 18 to 90 points.

*OMN* omnivorous; *VGN* vegan; *OVL* ovo-lacto-vegetarian; *PCV* pesco-vegetarian *PB* plant-based; *alt* alternative; *alc* alcohol

^a^n (%) for categorical indices; *p* value derived from Pearson Chi-square test; ^b^Fisher’s exact test

^c^ Median and interquartile range for continuous variables (BMI); *p* value derived from Kruskal–Wallis test

^d^ Dietary intakes in grams per day (adjusted per 1,000 kcal/day) are presented as means and standard deviations (SD) to facilitate interpretation and comparability with other studies. As the variables did not follow a normal distribution, the Kruskal–Wallis test was used for group comparisons. *P*-values were corrected for multiple testing by Benjamini-Hochberg

The second cluster, resembling an unhealthy OMN diet, comprised 142 OMN and 4 PBD followers. Participants of this cluster reported a high consumption of dairy products (133.1 g/d), meat (62.1 g/d), fish/seafood (31.8 g/d), refined cereals (35.1 g/d), pastries (20.8 g/d), sugars (7.7 g/d), non-alcoholic beverages (36.4 g/d), and OMN precooked meals (21.1 g/d). In contrast, healthy PB foods such as vegetables/mushrooms (120.3 g/d), legumes (26.7 g/d), fruits (136.7 g/d), and nuts (4.8 g/d) were less consumed than in the other clusters.

The third cluster, attributable to a healthy OMN diet, was composed of 396 OMN and fewer VGN and OVL/PCV (14, 3.4%). This cluster was characterized by a high intake of dairies (149.1 g/d), eggs (19.9 g/d), total meat (59.7 g/d), fish/seafood (34.5 g/d), whole grain cereals (21.0 g/d), and refined cereals (35.7 g/d).

Importantly, the subgroup of OMN included in first cluster exhibited a PB-like diet. As shown in Table 4, and confirmed by pairwise comparisons (Table S13), when compared to OMN of the other clusters, this specific group of OMN followed a PB-like diet and showed a significantly (*p* < 0.001) lower intake of milk (48.0 vs. > 95.2 g/d), red meat (13.9 vs. > 17.8 g/d), and processed meat (9.0 vs. > 14.0 g/d), refined cereals (24.5 vs. > 35.0 g/d), pastries (5.7 vs. > 8.7 g/d), and precooked meals (9.0 g/d vs. > 12.2 g/d), together with higher intake of vegetables (207.7 vs. < 110.6 g/d), fruits (201.3 vs. < 138.5 g/d), nuts (13.8 vs. < 8.3 g/d), whole grains (25.5 vs. < 20.8 g/d), coffee/tea (40.3 vs. < 26.0 g/d), spices (2.6 vs. < 1.9 g/d), and PB proteins (8.4 vs. < 2.4 g/d) and beverages (65.1 vs. < 7.9 g/d). Intake of fish, seafood and white meat, did not differ between the groups of OMN (*p* > 0.05).

**Table 4:** Description of omnivores classified in the three HCA clusters in the OMIVECA study

		Cluster 1: PBD n = 61	Cluster 2: Unhealthy OMN diet n = 142	Cluster 3: Mixed OMN diet n = 396	*p* value
Age^a^		21.0 (19.0;30.0)	21.0 (19.0;26.0)	22.0 (20.0;26.0)	0.481
Sex^b^	Female	51 (83.6%)	91 (64.1%)	285 (72.0%)	0.023
hPDI cat.^c^	High (≥ 65p)	23 (37.7%)	3 (2.1%)	52 (13.1%)	< 0.001
BMI^a^		21.5 (20.4;23.2)	22.8 (20.4;25.1)	22.5 (20.7;24.4)	0.215
Current smoker^c^	4 (6.6%)	14 (9.9%)	22 (5.6%)	0.357	
Physical activity^c^					< 0.001
	High	33 (54.1%)	31 (21.8%)	159 (40.2%)	
	Moderate	25 (41.0%)	81 (57.0%)	190 (48.0%)	
	Low	3 (4.9%)	30 (21.1%)	47 (11.9%)	
Milk^d^		48.0 (50.0)	95.2 (73.8)	98.5 (85.4)	< 0.001
Total dairy^d^		102.4 (65.5)	136.0 (76.9)	150.2 (94.7)	< 0.001
Egg^d^		17.8 (14.5)	12.5 (7.9)	19.6 (18.6)	< 0.001
Red meat^d^		13.9 (13.1)	21.6 (14.4)	17.8 (14.4)	< 0.001
White meat^d^		30.0 (26.6)	25.3 (16.5)	28.4 (20.1)	0.389
Processed meat^d^		9.0 (6.9)	15.8 (11.3)	14.0 (11.8)	< 0.001
Total meat^d^		53.5 (35.4)	63.8 (26.6)	61.4 (31.2)	0.015
Total fish & seafood^d^		35.9 (20.4)	32.1 (17.5)	35.0 (18.3)	0.206
Potatoes^d^		20.0 (20.5)	25.9 (17.5)	20.7 (14.1)	0.001
Vegetables^d^		207.7 (138.8)	110.6 (70.8)	161.3 (85.6)	< 0.001
Vegetables & mushrooms^d^		223.0 (141.0)	118.5 (73.7)	172.2 (90.3)	< 0.001
Legumes^d^		34.8 (21.9)	26.0 (16.8)	35.1 (23.7)	< 0.001
Fruits^d^		201.3 (124.3)	138.5 (80.6)	191.4 (125.5)	< 0.001
Nuts^d^		13.8 (13.1)	4.8 (4.5)	8.3 (7.1)	< 0.001
Whole grain cereal^d^		25.5 (20.2)	10.9 (13.2)	20.8 (21.2)	< 0.001
Refined cereal^d^		24.5 (19.1)	35.0 (19.0)	35.6 (25.4)	< 0.001
Pastries^d^		5.7 (5.8)	20.8 (17.8)	8.7 (7.3)	< 0.001
Snacks^d^		1.7 (2.5)	5.2 (5.6)	1.9 (1.8)	< 0.001
Vegetable fat^d^		9.3 (5.7)	9.3 (6.5)	10.3 (6.3)	0.143
Animal fat^d^		0.7 (3.1)	0.5 (0.9)	0.4 (0.9)	0.014
Sugars^d^		3.5 (4.6)	7.4 (6.7)	3.8 (3.7)	< 0.001
Coffee & tea^d^		40.3 (34.6)	24.3 (25.5)	26.0 (26.8)	0.002
Total non-alc. beverages^d^		15.4 (23.5)	36.8 (56.2)	13.4 (17.3)	< 0.001
Total alc. beverages^d^		20.0 (37.0)	32.1 (56.9)	15.1 (21.3)	0.044
Vegan precooked meals^d^		1.0 (2.5)	0.6 (2.9)	0.6 (1.8)	0.180
Omnivorous precooked meals^d^		8.1 (6.6)	21.7 (15.4)	11.6 (8.3)	< 0.001
Total precooked meals^d^		9.0 (7.4)	22.4 (16.2)	12.2 (8.7)	< 0.001
Sauces^d^		3.5 (3.4)	9.0 (7.2)	4.5 (3.9)	< 0.001
Soups & creams^d^		2.0 (4.3)	5.3 (7.3)	4.1 (7.7)	< 0.001
Spices^d^		2.6 (2.0)	1.6 (2.1)	1.9 (1.9)	< 0.001
Salt^d^		1.8 (1.4)	1.3 (1.1)	1.4 (1.0)	0.046
PB alt. protein^d^		8.4 (10.2)	1.6 (5.2)	2.4 (5.7)	< 0.001
Seaweed^d^		0.1 (0.3)	< 0.1 (0.1)	< 0.1 (0.1)	0.006
Vegan pastries^d^		0.7 (5.2)	0.2 (0.8)	0.1 (0.4)	0.413
Spreads^d^		3.0 (3.5)	2.0 (2.7)	2.3 (3.1)	0.172
PB beverages^d^		65.1 (70.9)	6.9 (25.6)	7.9 (20.9)	< 0.001
Smoothies^d^		9.4 (18.1)	1.9 (3.8)	1.5 (3.3)	0.002
Vegan dairy-like foods^d^		22.1 (31.2)	2.5 (9.1)	2.7 (7.4)	< 0.001

The food groups included were the 32 used to explore PCA. OMN: omnivorous; *PBD* Plant-based diet; *PB* plant-based; *alt* alternative; *alc* alcohol; *cat* categorized

^a^ Categorical variables are expressed as n (%) and analyzed using the Pearson chi-square test and ^c^Fisher’s exact test

^b^ Age and BMI are presented as median and intervale range and compared using the Kruskal–Wallis test

^d^ Dietary intakes (adjusted per 1000 kcal/day) are presented as means and standard deviations to facilitate the interpretation and comparability with other studies; they were compared using the Kruskal–Wallis test. *P* values were corrected for multiple testing by Benjamini-Hochberg

**Fig. 3:**
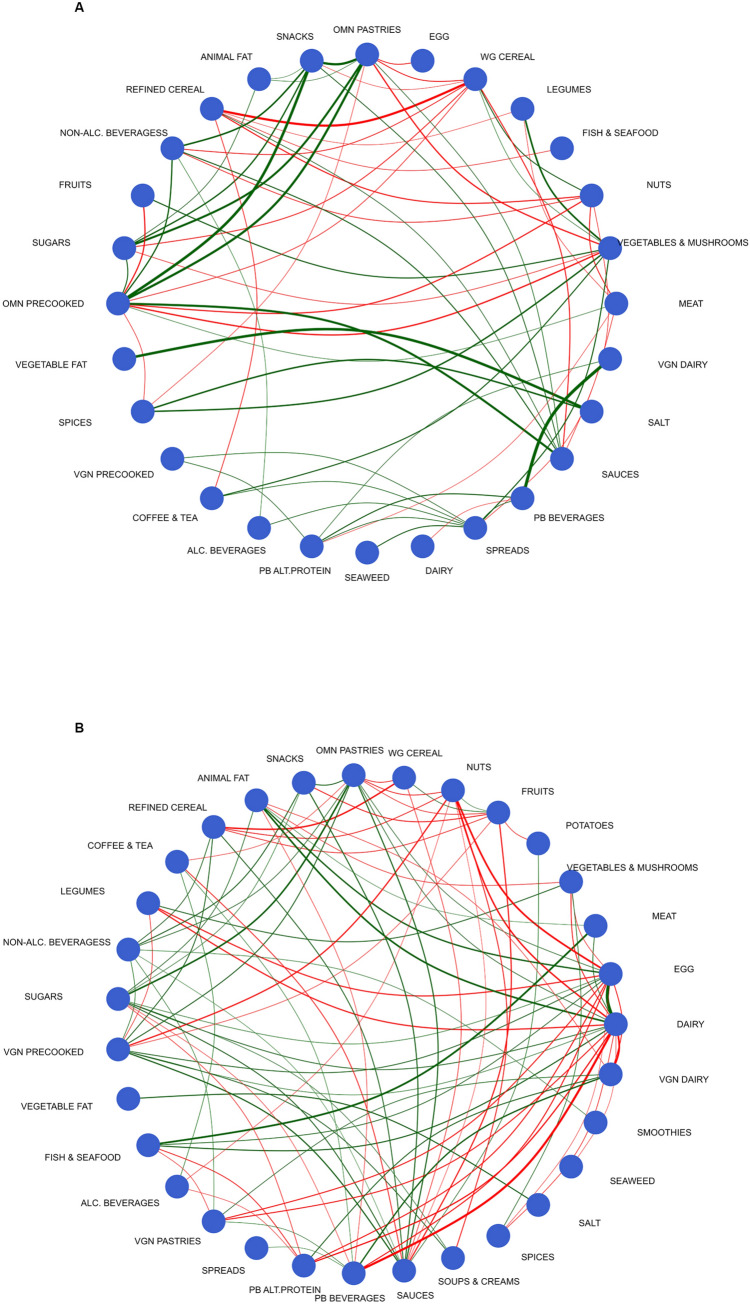
Network analysis. Correlations between the food groups. **A** Food group correlations in the OMN diet are shown for Spearman correlation coefficients greater than 0.3. Line thickness reflects the strength of the correlation; green lines indicate positive correlations, and red lines indicate negative ones. **B** Food group correlations in the grouped PBDs are shown for Spearman correlation coefficients greater than 0.3. Line thickness reflects the strength of the correlation; green lines indicate positive correlations, and red lines indicate negative ones. *Abbreviations* OMN: omnivorous; PBD: plant-based diet

By applying network food analysis, we were also able to visualize the interactions between different food groups across groups (Fig. 3). In participants following PBDs (Fig. 3A), positive correlations were observed between PB foods and vegetarian-like foods (legumes, vegetables, spices, PB beverages, etc.). In contrast, in the OMN (Fig. 3B), positive correlations were observed between animal-based foods (e.g., fish and meat) while negative correlations were noted between nuts, eggs, dairies, and refined cereals.

Overall, the above-described results did not change, or did to a low extent, when performing the sensitivity analysis described above regarding food intake by type of diet (data not shown) and patterns (Table S14).

## Discussion

The present study aimed to characterize PBD patterns using both diet index scores and clustering methods, by applying a priori and a posteriori approaches, to identify commonalities and differences in food intake compared to an OMN diet. The results show that dietary differences in intake go beyond the exclusion of animal-based foods. Participants following a PBD, including VGN, OVL and PCV, showed markedly higher intake of vegetables/mushrooms, legumes, and whole grain cereals, and a lower consumption of sugars than the OMN, independent of total energy intake. In contrast, the four diet groups showed similar levels of consumption of fruits and potatoes. Overall, both OVL and PCV showed similar dietary behaviors.

Our results also support that following a PBD is associated with higher scores in hPDI, as well as high adherence to the MD and planetary EAT-Lancet diet, with the VGN showing the highest adherence and the OVL and PVC exhibiting a similar adherence. A posteriori dietary pattern analyses including conventional approaches such as PCA and other innovative methods such as HCA, shed light on the different subgroups: a group of PBD including VGN, OVL and PCV, OMN with a PB-like behavior, OMN with mixed patterns, and as well as both OMN and some PBD followers with unhealthier dietary profiles.

### Intake of food groups across the PBD and non-PBD groups

Consistent with previous studies, our results confirm that individuals following a PBD consume higher levels of vegetables/mushrooms, legumes, and nuts [16]. This increase is driven not only by the need to substitute foods to meet energy requirements but also to achieve satiety [48, 49]. This would explain the difference in the aforementioned food groups, as well as the lack of difference in fruit and potato intake between groups [48]. Fruits and potatoes are neither substitution foods nor foods that need to be replaced in PBDs. The greater use of spices among VGNs compared to OMN, could be due to need of enhancing the flavor of vegetable foods and PB dishes [50]. Regarding beverages, our results highlight important differences in milk consumption and PB beverages (as milk alternatives) across the diet groups. More specifically, VGNs consumed as much PB beverage as OMN combining both milk and PB milk alternatives. Interestingly, OVL and PCV, despite being allowed to consume milk, reported similar intakes of both milk and PB alternatives. This novel result has not been reported previously, likely due to the lack of information on the consumption of PB beverages in earlier studies.

It is important to acknowledge that this differing food intake profiles may have nutritional implications. While this was not evaluated in the present study, several studies have reported some nutritional shortfalls in PBDs [16, 48, 51–54], including in particular a systematic review of 141 studies that evaluated nutritional differences between PBD and non-PBD [16]. The main nutritional differences relate to fiber, proteins, polyunsaturated fatty acids (PUFAs) and Saturated fatty acids (SFAs), and selected vitamins and minerals (iron, iodine, vitamin B12, etc.). In fact, the dietary source providing proteins is not the same: VGN people replace fish and meat (protein source for OMN), by vegetable protein from legumes, nuts, cereals and PB alternative proteins. Among vegetarians, PCV eat fish and consume as much eggs as OVL. In fact, in our study, both dietary patterns shared similar levels of intake, except for fish intake. However, the dietary protein quality of PBDs is presumed to be lower because PB foods contain limited amounts of certain amino acids (methionine, cysteine, and lysine) [55]. Similarly, PBDs provide iron, although with limited bioavailability due to the low absorption of non-heme iron from PB foods [56]. The most favorable aspect of PBDs is that PB foods (vegetables, fruits and whole grains) contribute to higher fiber intake, lower SFA intake, and higher PUFA intake, owing to the replacement of meat and meat products by fish, nuts and seeds [16, 57]. However, among the main drawbacks, because of the avoidance of animal foods in strict PBDs, lower vitamin B12 intake and status are likely [14].

### Dietary PB indices

The overall high adherence of the oPDI among VGN, followed by OVL, PCV was expected due to the restriction of animal-based foods in PBDs. Likewise, a PBD adherence was associated with higher scores in the other indices reflecting PBD (MD score and EAT-Lancet index). Adherence to these indices has consistently been related to multiple health benefits [22, 58–60], suggesting that following a PBD may imply a better health status. Previous studies evaluating adherence to diet quality indexes (to the MD and Healthy Eating Index) also concluded that PBDs followers, both VGN and vegetarians, showed a better adherence compared to OMN [54]. Despite the conceptual overlap between PBDs and the PDI, adherence to the PDI among individuals following PBDs has not been specifically assessed. Nevertheless, the interpretation of the uPDI within PBD group is particularly complex because animal-based foods score negatively in this index. Therefore, assessing adherence to the uPDI in PBD diets is challenging. Likewise, for the MD score, due to the dietary restrictions in PBDs it is difficult to assign positive points to certain favorable components, such as fish for VGN and OVL. As a result, the uPDI and MD score showed limited discriminatory capacity for PBDs in our study. This limitation highlights the need to refine existing indices to better capture PBD patterns, ensuring a more balanced inclusion of alternative food sources, as previously noted by Clarys et al. [54].

### A posteriori methods

Three main dietary patterns were identified in our study using a posteriori methods. The explained variance in the components retained in the PCA analyses (~ 28%) was similar to that reported in other studies in nutritional epidemiology [61]. PCA analysis has previously been used to characterize VGN and vegetarian diets [62–64], yet not across the spectrum of all possible groups of VGN, vegetarians and OMN. For example, in a German cross-sectional study of 110 VGN, 145 vegetarians and 135 OMN conducted in children and adolescents, three patterns were identified, two of which were related to PB food consumption (“Vegetables and fruits”, “meat alternatives and potatoes”) [64].

Another German study of 516 VGN, identified two dietary patterns through PCA, labeled “convenience” and “health-conscious” [62]. Similarly, in the EPIC-Norfolk study, four patterns were retrieved through PCA and cluster analyses from a study sample of 129 VGN. Within these patterns, both healthy and unhealthy patterns were also detected [63]. The latter two studies were the first to highlight a group of VGN following a “convenience” dietary pattern with increased intake of processed foods and VGN products. In the present study, these findings could not be confirmed due to the limited number of VGN participants (N = 57). Instead, we performed PCA across all the PB and non-PBD groups, complemented with a cluster analyses using different techniques to further explore dietary patterns. While the combination of PCA and cluster analysis has been proposed to improve and validate dietary pattern characterization [65], it has not been applied in the context of PBDs. Among the advantages of these a posteriori methods is the possibility of comparing our findings with those of previous studies, particularly with PCA, widely used in nutritional epidemiology. In addition, PLS-DA, as a supervised method, complements PCA by allowing us to assess whether the main direction of the analyses is independent of the self-reported dietary group. Furthermore, t-SNE allows a proper visualization of the data. Regarding clustering approaches, k-means, characterizes the dietary patterns identified after PCA dimensional reduction further. Finally, Ward’s hierarchical clustering method allows a more detailed evaluation of similarities and differences between individuals, facilitating identification of gradients across dietary patterns. The combination enabled us to elucidate shared dietary habits among PBD followers, as well as to distinguish key differences. Together, PCA and all the other clustering techniques showed, to some extent, three main clusters: a PBD-like pattern, an unhealthy pattern, and a mixed animal and PBD pattern. Also, our study shows that, in general, different PBD-like patterns converge into a common PB dietary pattern. In line with the aforementioned studies, the present study supports that a subgroup of PBD followers have less healthy dietary habits, characterized by higher intakes of precooked foods and added sugars. In addition, we identified a subgroup of OMN following a more PB-like diet, which clustered with the PBD group. This subgroup of OMN showed a higher intake of vegetables and fruits and a moderate consumption of animal foods. Furthermore, its adherence to the hPDI lay between the OVL and PCV groups (OR = 4.2, data not shown), which also reinforces that this group of OMN and the PBD groups are closely related. Although this PB-like OMN does not meet the definition of the flexitarian diet (consuming at least once per month but less than once per week animal food) [66], this pattern could make more acceptable PBDs to a wider range of consumers due to its lower level of restriction compared with vegetarian or VGN diets. For a 2000 kcal diet, the recommended intake for this subgroup would be ≤ 1 serving/week of red meat (< 28 g/day), ≥ 4 servings/day of fruits and vegetables (> 800 g/day), and ≥ 1 serving/day of whole grains (> 50 g/d).

### Strengths and limitations

The main limitation of this study is misreporting associated with the use of a FFQ [67]. Potential outliers of energy intake based on percentile criteria were excluded to mitigate this source of bias; however, residual misreporting cannot be ruled out. Furthermore, the study population consisted primarily of young university students in health-related fields, which may minimize possible information bias, and women. While this fact could limit the extrapolation of our findings, sensitivity analysis stratified by sex, age and profession/degree yielded comparable results. In addition, the robustness of our findings is supported by the use of a validated FFQ and that dietary information derived from 24-h recalls (for > 200 participants) yielded similar results.

This study also presents some important strengths. First, our FFQ was specifically adapted to PBD, including 31 specific food items such as meat alternatives, VGN cheese, PB beverages/milk, and other PB foods, thereby providing a proper assessment of these groups. This enabled us to detect differences in dietary behaviors apart from the restriction in meat consumption. Second, to our knowledge, we are the first to evaluate both adherence to a PBD and characteristics of the dietary patterns in PBD and non-PBD populations. Third, since stability of the patterns derived by data-driven methods (especially cluster analysis) is a concern, the stability of the derived a posteriori dietary patterns was confirmed in the sensitivity analysis. Fourth, by the use of different clustering methods we could verify that VGN and vegetarians, as well as a subgroup of OMN with a PB-like pattern, behave similarly regarding dietary intake.

## Conclusion

The current study supports that there are differences in food intake between OMN and VGN, OVL, and PCV, beyond the exclusion of foods of animal origin. By applying both a priori and a posteriori approaches, we identified distinctive dietary profiles and similarities among the diet groups studied. PBD groups cluster together, with OVL and PCV diets being the most similar in terms of food intake. Also, this study uncovers novel dietary profiles: e.g., a subgroup of OMN followed PB-like dietary patterns driven by reduced consumption of animal foods, while not all PBD followers adhered to healthy eating practices. Further research is needed to better characterize both healthy OMN and less healthy PBD for guiding sustainable eating transitions.

## Supplementary Information

Below is the link to the electronic supplementary material.

Supplementary file1 (DOCX 285 KB)

## Data Availability

Data will be made available on request and subject to approval by the relevant ethics committee.

## Abbreviations

BMI: Body mass index
CI: Confidence intervals
FL: Factor loadings
FFQ: Food frequency questionnaire
hPDI: Healthy plant-based diet index
HCA: Hierarchical clustering analysis
IQR: Interquartile range
ISCIII: Institute of health Carlos III
MD: Mediterranean diet
OR: Odds ratio
OMN: Omnivorous/omnivore
OVL: Ovo-lacto-vegetarians
PLS-DA: Partial least squares discriminant analysis
PCV: Pesco-vegetarians
PB: Plant-based
PBD: Plant-based diet
PA: Physical activity
PUFA: Polyunsaturated fatty acids
PCA: Principal component analysis
PDI: Plant-based diet index
oPDI: Overall plant-based diet index
SFA: Saturated fatty acids
SD: Standard deviation
t-SNE: t-distributed Stochastic Neighbor Embedding
uPDI: Unhealthy plant-based diet index
VIP: Variable importance in projection
VGN: Vegan
