# Supplementary material for: Omnivorous and plant-based dietary patterns: a comparative analysis using data-driven and index-based approaches
Source: Eur J Nutr. 2026 Jul 11;65(5):207. doi: 10.1007/s00394-026-04046-z (PMC13356088; doi:10.1007/s00394-026-04046-z)
Supplement: Supplementary file 1 — Supplementary file1 (DOCX 285 KB) [file 394_2026_4046_MOESM1_ESM.docx]

SUPPLEMENTAL MATERIAL

**TABLES**

**Table S1:** Items included in the original and modified food frequency questionnaire, grams per portion and factor correction for seasonality.

**Table S2:** Description of food groups: 19 General Foods Groups and 65 total groups.

**Table S3:** Provegetarian diet indices components (18 components).

**Table S4:** EAT-Lancet scoring scheme.

**Table S5:** Food groups used in a posteriori methods (32 items).

**Table S6.** Dietary intake of the main food groups by type of diet in the OMIVECA study.

**Table S7.** Post-hoc analysis of the dietary intake of the main food groups by type of diet in the OMIVECA study.

**Table S8.** Raw dietary intake of the main food groups by type of diet in the OMIVECA study.

**Table S9.** Logistic regression model. Adherence to high hPDI by diet, adjusted.

**Table S10:** Factor loadings of each variable in PCA in the OMIVECA study.

**Table S11:** Description of food groups by PCA clusters in the OMIVECA study.

**Table S12:** Factor loadings and VIP of the three first components in PLS-DA in the OMIVECA study.

**Table S13:** Post-hoc analysis of omnivores classified in the three HCA clusters in the OMIVECA study.

**Table S14:** Factor loadings derived from PCA of the four main sensitivity analyses in the OMIVECA study.

**FIGURES**

**Figure S1:** Adherence high hPDI bivariate model plot by type of diet.

**Figure legend**: The boxplot shows the distribution of the healthy provegetarian diet index (hPDI) scores by dietary group. P-values were obtained from bivariate logistic regression analyses where hPDI was categorized into “High” (≥ 65p) and “Low” (>65p). Black dots represent individual hPDI scores as continuous values.

**Table S1:** Grams per portion and factor correction for seasonality in the original food frequency questionnaire and added items.

| **Added items** | | |
| --- | --- | --- |
| Food item | Grams per portion | Information |
| Corn | 82 | Boiled |
| Other spices (curcuma, etc.) | 2 | - |
| Berries | 100 | - |
| Avocado | 50 | - |
| Dark chocolate >70% | 20 | - |
| Pre-cooked meals (lasagna, etc.) | 150 | - |
| Ketchup sauce | 10 | - |
| Non-alcoholic beer | 330 | - |
| Brown rice | 60 | Raw |
| Whole wheat pasta | 60 | Raw |
| Gazpacho out of season | - | Yes/No |
| Iodized salt | - | Yes/No |
| Brown rice | - | Yes/No |
| Whole wheat pasta | - | Yes/No |
| Tea | - | Type: Black, green, other infusions |
| Sweeteners | - | Type: Sugar, agave, panela, maple (syrup), cane honey, none |
| Plant-based beverages | - | Ingredients: Sugar, no added sugar, calcium, vitamins (B12, D, etc.), unknown |
| **Original FFQ** |  |  |
| Food item | Grams per portion | Seasonality factor correction |
| Orange, grapefruit or two tangerines | 175 | 0.8 |
| Cabbage, cauliflower, and broccoli | 200 | 0.5 |
| Andalusian gazpacho | 200 | 0.25 |
| Ice cream | 75 | 0.25 |
| Strawberries | 150 | 0.25 |
| Vegan ice cream | 75 | 0.25 |
| Watermelon | 225 | 0.25 |
| Cherries, picota cherries, and plums | 150 | 0.17 |
| Nougat and marzipan | 90 | 0.08 |

**Table S2.** Description of food groups: 19 General Foods Groups and 65 total groups.

| **General Foods Groups** | **Groups' Components** |
| --- | --- |
| **DAIRY** | **MILK**: Whole milk, semi-skimmed milk, skimmed milk, condensed milk, milkshakes |
|  | **OTHER DAIRY**: Low-fat yogurt, whole yogurt, petit-suisse, curd, white cheese, portioned cheese, other cheeses, cream, and kefir |
|  | **DAIRY DESSERTS**: Custards, ice cream |
|  | **TOTAL DAIRY**: MILK, OTHER DAIRY, AND DAIRY DESSERTS |
| **EGG** | **EGG**: Eggs |
| **MEAT AND DERIVATES** | **RED MEAT**: Beef, lamb, pork |
|  | **WHITE MEAT**: Chicken/turkey with skin, chicken/turkey without skin, rabbit |
|  | **PROCESSED MEAT**: Processed meats, Spanish ham, cooked ham, bacon, hamburgers |
|  | **OFFAL AND PÂTÉS**: Offal, liver, and pâtés |
|  | **TOTAL MEAT**: RED MEAT, WHITE MEAT, PROCESSED MEAT, OFFAL, AND PÂTÉS |
| **FISH AND SEAFOOD** | **WHITE FISH**: White fish, salted fish |
|  | **BLUE FISH**: Blue fish, canned natural fish, fish in oil |
|  | **CANNED FISH**: Canned natural fish, fish in oil |
|  | **SEAFOOD**: Oysters/clams, crustaceans, squid/cuttlefish |
|  | **TOTAL FISH AND SEAFOOD**: WHITE FISH, BLUE FISH, CANNED FISH, AND SEAFOOD |
| **VEGETABLES AND MUSHROOMS** | **POTATOES**: Boiled potatoes, fried potatoes |
|  | **VEGETABLES**: Spinach/chard, cabbage/cauliflower/broccoli, garlic, onion, carrot/pumpkin, green beans, eggplant/cucumber/zucchini, peppers, artichoke/leek/celery, asparagus, lettuce/escarole/endives, tomato |
|  | **MUSHROOMS**: Wild mushrooms/button mushrooms/etc. |
|  | **VEGETABLES AND MUSHROOMS**: VEGETABLES, MUSHROOMS |
| **LEGUMES** | **LEGUMES**: Lentils, beans, chickpeas, peas/broad beans, hummus |
| **FRUITS** | **FRUITS:** Orange/lemon, banana, apple/pear, strawberries, cherries/plums, peach/apricot, watermelon, melon, kiwi, grapes, berries, avocado, orange juice, natural fruit juice, canned fruits, dates/figs, olives |
| **NUTS** | **NUTS**: Seeds, almonds/peanuts, walnuts |
| **CEREALS** | **WHOLE GRAIN CEREALS**: Whole grain breakfast cereals, whole wheat bread, quinoa, brown rice, whole wheat pasta |
|  | **REFINED CEREALS**: Breakfast cereals, white rice, white bread, pasta, corn, couscous |
|  | **SUM OF CEREALS**: WHOLE GRAINS, REFINED CEREALS |
| **SNACKS AND PASTRIES** | **SNACKS**: Snacks, commercial chips |
|  | **PASTRIES**: Chocolate cookies, pastries, croissant/ensaimada, donuts, muffins, cakes, churros**,** whole wheat cookies |
|  | **VEGAN PASTRIES**: Vegan cookies/pastries, vegan baked goods |
|  | **TOTAL PASTRIES:** PASTRIES, VEGAN PASTRIES |
| **SUGARS** | **SUGARS**: Chocolate/bonbons, nougat, shortbread/marzipan, sugar, honey, cocoa powder, jams |
|  | **DARK CHOCOLATE**: Dark chocolate |
| **FATS** | **OLIVE OIL**: Extra virgin olive oil, olive oil, pomace olive oil |
|  | **VEGETABLE FATS**: OLIVE OIL, sunflower oil, corn oil, soybean oil, other fats (coconut), margarine |
|  | **ANIMAL FATS**: Butter, pork/cow lard |
| **BEVERAGES** | **COFFEE AND TEA**: Coffee, decaffeinated coffee, tea, kombucha |
|  | **SUGARY DRINKS**: Carbonated sugary drinks |
|  | **SWEETENED DRINKS**: Sugar-free drinks |
|  | **BOTTLED JUICES**: Fruit juice, grape must |
|  | **TOTAL NON-ALCOHOLIC BEVERAGES**: SUGARY DRINKS, SWEETEND DRINKS, BOTTLED JUICES |
|  | **PLANT-BASED BEVERAGES**: Oat drink, almond drink, soy drink, other vegan beverages |
| **ALCOHOL** | **WINES**: Young red wine, aged red wine, rosé, muscat, white wine, cava |
|  | **BEER**: Beer |
|  | **SPIRITS**: Aniseed liquors, distilled spirits |
|  | **NON-ALCOHOLIC BEER**: Non-alcoholic beer |
|  | **TOTAL ALCOHOLIC BEVERAGES**: WINES, BEER, DISTILLED SPIRITS |
| **SPICES** | **SPICES**: Parsley/thyme/bay leaf, spicy seasonings, turmeric, other spices |
|  | **SALT**: Salt |
| **PRECOOKED** | **PRECOOKED MEALS**: Cannelloni/lasagna, croquettes, pizza |
|  | **VEGAN PRECOOKED MEALS**: Fried vegan precooked foods, vegan precooked pasta, vegan pizza  **SUM OF PRECOOKED:** PRECOOKED MEALS, VEGAN PRECOOKED MEALS |
| **PREPARED** | **SAUCES**: Mayonnaise, ketchup, mustard, fried tomato |
|  | **SOUPS AND CREAMS**: Soups and creams |
|  | **PREPARED MEALS**: PRECOOKED, VEGAN PRECOOKES, SAUCES, SOUPS AND CREAMS |
| **VEGETARIAN/VEGAN FOODS** | **PLANT-BASED ALERNATIVE PROTEIN**: Tofu, seitan, tempeh, textured soy, other proteins, cereal-based meat, legume-based meat, soy meat |
|  | **MEAT ALTERNATIVES**: Cereal-based meat, legume-based meat, soy meat |
|  | **SEAWEED**: Seaweed |
|  | **SPREADS**: Vegan pâtés, vegan spreadable cheese, hummus, guacamole |
|  | **KOMBUCHA**: Kombucha |
|  | **SMOOTHIES**: Vegetable smoothies |
| **VEGAN DAIRY-LIKE** | **VEGAN CHEESE**: Vegan cheese substitute |
|  | **VEGAN YOGURT**: Soy-based dairy alternatives, other plant-based dairy alternatives |
|  | **VEGAN ICE CREAM**: Vegan ice cream |
|  | **VEGAN DAIRY-LIKE FOODS**: VEGAN CHEESE, VEGAN YOGURT, VEGAN ICE CREAM |

**Table S3.** Plan-based diet indices components (18 components).

| Components | | oPDI | hPDI | uPDI |
| --- | --- | --- | --- | --- |
| **Plant-based foods** | |  |  |  |
|  | Vegetables & mushrooms | **+** | **+** | **-** |
|  | Legumes | **+** | **+** | **-** |
|  | Fruits | **+** | **+** | **-** |
|  | Nuts | **+** | **+** | **-** |
|  | Whole grain cereal | **+** | **+** | **-** |
|  | Vegetable fat | **+** | **+** | **-** |
|  | Coffee & tea | **+** | **+** | **-** |
|  | Bottled juices | **+** | **-** | **+** |
|  | Refined cereal | **+** | **-** | **+** |
|  | Potatoes | **+** | **-** | **+** |
|  | Sugary drinks | **+** | **-** | **+** |
|  | Pastries | **+** | **-** | **+** |
| **Animal-based foods** | |  |  |  |
|  | Dairy | **-** | **-** | **-** |
|  | Egg | **-** | **-** | **-** |
|  | Meat | **-** | **-** | **-** |
|  | Fish | **-** | **-** | **-** |
|  | Precooked meals * | **-** | **-** | **-** |
|  | Animal fat | **-** | **-** | **-** |
| oPDI: overall plant-based diet index; hPDI: healthy plant-based diet index; uPDI: unhealthy plant-based diet index.  * Plant-based precooked meals score positively to oPDI index and Precooked meals (omnivorous) score reversely. For uPDI index, plant-based precooked meals group is another component of the index (19 components). | | | | |

**Table S4.** EAT-Lancet scoring scheme.

| **Dietary Components** | **Criteria for Scoring 1 Point** | **Food Items (FFQ)** |
| --- | --- | --- |
| **Whole grains** | ≤464g/day and whole grain fiber >5g | Whole grain breakfast cereals, whole wheat bread, quinoa, brown rice, whole wheat pasta, breakfast cereals, white rice, white bread, pasta, corn, couscous |
| **Tubers and starchy vegetables** | ≤100g/day | Boiled potatoes, fried potatoes, commercial chips |
| **Vegetables** | ≥200g/day | Spinach/chard, cabbage/cauliflower/broccoli, garlic, onion, carrot/pumpkin, green beans, eggplant/cucumber/zucchini, peppers, artichoke/leek/celery, asparagus, lettuce/escarole/endives, tomato, wild mushrooms/button mushrooms |
| **Fruits** | ≥100g/day | Orange/lemon, banana, apple/pear, strawberries, cherries/plums, peach/apricot, watermelon, melon, kiwi, grapes, berries, avocado |
| **Dairy foods** | ≤500g/day | Whole milk, semi-skimmed milk, skimmed milk, condensed milk, milkshakes, low-fat yogurt, whole yogurt, petit-suisse, curd, white cheese, portioned cheese, other cheeses, cream, and kefir custard, ice cream |
| **Protein sources** |  |  |
| – Beef, lamb, pork | ≤28g/day | Beef, lamb, pork |
| – Chicken, other poultry | ≤58g/day | Chicken/turkey with skin, chicken/turkey without skin, rabbit |
| – Eggs | ≤25g/day | Eggs |
| – Fish | ≤100g/day | White fish, salted fish, blue fish, canned natural fish, fish in oil, oysters/clams, crustaceans, squid/cuttlefish |
| **Legumes** |  |  |
| – Dry beans, lentils, peas | ≤100g/day | Lentils, beans, chickpeas, peas/broad beans, hummus |
| – Soy foods | ≤50g/day | Tofu, textured soy, soy meat |
| – Peanuts or tree nuts | ≥25g/day | Seeds, almonds/peanuts, walnuts |
| **Added fats** | Ratio of 0.8 for unsaturated: saturated fat intake | MUFA (g) / SFA (g) |
| **Added sugars** | ≤31g/day | Chocolate/bonbons, nougat, shortbread/marzipan, sugar, honey, cocoa powder, jams |

Energy adjustment was 2,000 kcal for women and 2,500 kcal for men.

FFQ: Food Frequency Questionnaire; SFA: saturated fatty acids; MUFA: monounsaturated fatty acids.

**Table S5.** Food groups used in a posteriori methods (32 items).

| Total dairy |
| --- |
| Egg |
| Total meat |
| Total fish & seafood |
| Potatoes |
| Vegetables & mushrooms |
| Legumes |
| Fruits |
| Nuts |
| Whole grain cereal |
| Refined cereal |
| Pastries |
| Snacks |
| Vegetable fat |
| Animal fat |
| Sugars |
| Coffee & tea |
| Total non-alc. beverages |
| Total alc. beverages |
| Vegan precooked meals |
| Omnivorous precooked meals |
| Sauces |
| Soups & creams |
| Spices |
| Salt |
| PB alt. protein |
| Seaweed |
| Vegan pastries |
| Spreads |
| PB beverages |
| Smoothies |
| Vegan dairy-like foods |

**Table S6.** Dietary intake of the main food groups by type of diet in the OMIVECA study.

|  | **All**  **n=760** | **OMN**  ***n=599*** | **PCV**  ***n=31*** | **OVL**  ***n=73*** | **VGN**  ***n=57*** | **p-value** |
| --- | --- | --- | --- | --- | --- | --- |
| Milk | 78.5 (80.7) | 92.6 (81.2) | 46.6 (67.3) | 37.5 (59.0) | 0.0 (0.0) | <0.001 |
| Other dairy | 42.1 (35.3) | 46.6 (34.1) | 37.3 (24.9) | 39.6 (40.6) | 0.2 (1.1) | <0.001 |
| Dairy desserts | 2.4 (4.4) | 2.7 (4.8) | 1.4 (2.1) | 1.8 (2.8) | 0.1 (0.5) | <0.001 |
| **Total dairy** | 123.0 (94.1) | 142.0 (89.2) | 85.3 (82.3) | 79.0 (82.6) | 0.3 (1.2) | <0.001 |
| **Egg** | 16.5 (16.5) | 17.7 (16.6) | 15.3 (8.2) | 19.8 (18.6) | 0.1 (0.3) | <0.001 |
| Red meat | 14.5 (14.8) | 18.3 (14.4) | 1.9 (6.3) | 0.1 (0.6) | 0.0 (0.0) | <0.001 |
| White meat | 22.1 (21.1) | 27.8 (20.1) | 2.6 (5.8) | 0.2 (0.7) | 0.0 (0.0) | <0.001 |
| Processed meat | 11.1 (11.6) | 13.9 (11.4) | 1.7 (4.7) | 0.3 (2.0) | 0.0 (0.0) | <0.001 |
| **Total meat** | 48.5 (36.8) | 61.2 (30.7) | 6.7 (17.1) | 0.5 (3.0) | 0.0 (0.0) | <0.001 |
| White fish | 8.3 (8.8) | 9.9 (8.6) | 10.8 (10.5) | 0.1 (0.6) | 0.0 (0.0) | <0.001 |
| Blue fish | 12.8 (11.4) | 15.4 (10.8) | 16.3 (13.2) | 0.7 (2.9) | 0.1 (0.5) | <0.001 |
| Canned fish | 4.3 (4.7) | 5.1 (4.7) | 5.3 (4.4) | 0.2 (0.8) | 0.0 (0.0) | <0.001 |
| Seafood | 7.7 (8.8) | 9.1 (7.9) | 13.9 (18.9) | 0.1 (0.8) | 0.1 (0.4) | <0.001 |
| **Total fish & seafood** | 28.9 (22.0) | 34.4 (18.4) | 40.9 (36.0) | 0.9 (3.4) | 0.1 (0.7) | <0.001 |
| **Potatoes** | 21.1 (15.4) | 21.8 (15.9) | 17.8 (13.0) | 19.4 (13.3) | 17.7 (13.5) | 0.081 |
| Vegetables | 166.1 (97.2) | 154.0 (93.4) | 195.9 (86.1) | 188.9 (62.1) | 248.1 (127.4) | <0.001 |
| Mushrooms | 12.5 (15.4) | 10.6 (14.3) | 18.0 (18.1) | 21.1 (20.0) | 18.0 (13.7) | <0.001 |
| **Vegetables & mushrooms** | 178.6 (102.1) | 164.7 (97.7) | 213.8 (89.6) | 209.9 (72.6) | 266.1 (127.8) | <0.001 |
| **Legumes** | 37.9 (25.1) | 32.9 (22.4) | 48.2 (23.9) | 51.2 (21.2) | 67.7 (29.4) | <0.001 |
| **Fruits** | 181.8 (116.4) | 179.9 (118.4) | 192.1 (80.2) | 196.3 (117.0) | 177.4 (111.8) | 0.219 |
| **Nuts** | 9.2 (8.8) | 8.0 (7.8) | 10.2 (8.0) | 10.3 (7.5) | 19.2 (12.8) | <0.001 |
| Whole grain cereal | 20.4 (19.9) | 19.0 (20.0) | 29.8 (20.4) | 24.7 (19.0) | 25.2 (16.2) | <0.001 |
| Refined cereal | 32.1 (22.8) | 34.3 (23.6) | 25.0 (17.3) | 25.6 (17.5) | 21.0 (16.3) | <0.001 |
| **Sum of cereals** | 52.5 (24.8) | 53.3 (26.1) | 54.8 (19.8) | 50.3 (18.8) | 46.2 (17.9) | 0.341 |
| Snacks | 2.6 (3.4) | 2.7 (3.5) | 2.3 (2.7) | 2.7 (2.6) | 2.5 (3.4) | 0.374 |
| Pastries | 10.3 (11.4) | 11.3 (11.9) | 8.3 (10.3) | 8.7 (8.3) | 3.9 (4.8) | <0.001 |
| Vegan pastries | 0.6 (2.4) | 0.2 (1.8) | 0.8 (1.8) | 1.6 (3.2) | 3.4 (4.1) | <0.001 |
| **Total pastries** | 10.8 (11.5) | 11.3 (12.0) | 8.94 (10.7) | 10.2 (9.6) | 7.05 (7.1) | 0.023 |
| **Sugars** | 4.2 (4.7) | 4.6 (4.9) | 3.3 (4.1) | 3.3 (4.0) | 1.8 (2.9) | <0.001 |
| Dark chocolate | 2.0 (3.1) | 1.9 (2.9) | 3.2 (4.6) | 2.4 (2.9) | 2.1 (3.8) | 0.081 |
| Olive oil | 9.5 (6.3) | 9.3 (6.3) | 9.2 (5.9) | 10.9 (6.5) | 9.6 (7.1) | 0.179 |
| Vegetable fat | 10.1 (6.4) | 9.9 (6.3) | 9.6 (5.7) | 11.5 (6.5) | 10.9 (7.6) | 0.171 |
| Animal fat | 0.4 (1.2) | 0.4 (1.3) | 0.3 (0.5) | 0.2 (0.5) | 0.0 (0.0) | <0.001 |
| **Coffee & tea** | 29.0 (28.7) | 27.1 (27.7) | 40.6 (41.6) | 37.1 (28.9) | 32.1 (26.8) | <0.001 |
| Sugary drinks | 6.3 (16.6) | 7.1 (18.3) | 4.6 (8.3) | 3.1 (5.2) | 3.7 (8.4) | 0.111 |
| Sweetened drinks | 7.5 (21.1) | 8.4 (23.2) | 3.4 (7.4) | 5.0 (11.2) | 3.4 (9.7) | 0.057 |
| Bottled juices | 3.2 (10.7) | 3.6 (11.6) | 0.6 (1.6) | 1.7 (5.2) | 2.4 (9.0) | 0.098 |
| **Total non-alc. beverages** | 17.1 (30.4) | 19.1 (33.1) | 8.6 (10.8) | 9.8 (15.3) | 9.5 (16.6) | <0.001 |
| **PB beverages** | 27.0 (55.3) | 13.5 (35.4) | 65.5 (75.5) | 58.2 (70.2) | 108.5 (89.1) | <0.001 |
| Wine | 3.5 (8.3) | 3.4 (8.2) | 4.9 (8.1) | 4.0 (10.3) | 2.8 (7.5) | 0.183 |
| Beer | 15.8 (31.4) | 15.3 (31.7) | 13.0 (15.5) | 23.4 (38.0) | 13.0 (25.1) | 0.197 |
| Spirits | 0.9 (1.9) | 1.0 (2.0) | 0.4 (0.7) | 0.4 (0.9) | 0.3 (0.8) | <0.001 |
| Non-alcoholic beer | 3.0 (11.8) | 2.3 (10.4) | 9.0 (18.9) | 4.7 (12.5) | 4.3 (17.4) | <0.001 |
| **Total alc. beverages** | 20.1 (35.0) | 19.7 (35.4) | 18.3 (20.2) | 27.8 (39.9) | 16.1 (29.8) | 0.069 |
| Spices | 2.2 (2.2) | 1.9 (2.0) | 2.5 (1.9) | 2.8 (1.8) | 4.2 (3.4) | <0.001 |
| Salt | 1.4 (1.1) | 1.4 (1.1) | 1.8 (1.4) | 1.6 (1.2) | 1.5 (1.1) | 0.179 |
| Omnivorous precooked meals | 10.8 (11.5) | 13.7 (11.3) | 0.0 (0.0) | 0.0 (0.0) | 0.0 (0.0) | <0.001 |
| Vegan precooked meals | 2.7 (6.2) | 0.6 (2.2) | 12.3 (10.1) | 12.4 (9.8) | 7.1 (6.7) | <0.001 |
| **Sum of precooked meals** | 13.5 (11.3) | 14.3 (11.7) | 12.3 (10.1) | 12.4 (9.8) | 7.1 (6.7) | <0.001 |
| Sauces | 5.2 (5.0) | 5.4 (5.2) | 3.7 (3.9) | 4.7 (4.5) | 3.5 (3.6) | 0.005 |
| Soups & creams | 3.9 (7.4) | 4.2 (7.4) | 2.5 (5.4) | 4.0 (8.6) | 1.7 (5.7) | 0.002 |
| **Prepared meals** | 23.2 (18.3) | 23.7 (18.4) | 22.9 (17.5) | 24.9 (18.6) | 15.4 (15.8) | <0.001 |
| Meat alternatives | 5.1 (12.2) | 1.3 (3.8) | 15.8 (18.5) | 19.1 (18.4) | 21.6 (21.9) | <0.001 |
| **PB alt. protein** | 13.9 (28.6) | 2.8 (6.5) | 33.9 (33.0) | 48.2 (32.2) | 76.2 (41.4) | <0.001 |
| Seaweed | 0.1 (0.2) | 0.1 (0.1) | 0.2 (0.4) | 0.1 (0.2) | 0.2 (0.2) | <0.001 |
| **Spreads** | 2.8 (3.5) | 2.3 (3.1) | 3.3 (3.1) | 5.1 (4.0) | 5.2 (4.5) | <0.001 |
| Kombucha | 1.2 (5.5) | 1.0 (5.3) | 1.0 (2.2) | 1.5 (3.4) | 3.5 (9.4) | <0.001 |
| Smoothies | 2.7 (7.7) | 2.4 (7.0) | 1.0 (2.5) | 4.1 (10.3) | 5.2 (11.5) | 0.057 |
| **Vegan dairy-like foods** | 7.3 (17.0) | 4.6 (13.7) | 11.9 (20.2) | 15.2 (23.5) | 22.9 (23.6) | <0.001 |

Dietary intakes in grams per day (adjusted per 1,000 kcal/day) are presented as means and standard deviations (SD) to facilitate interpretation. As the variables did not follow a normal distribution, the Kruskal-Wallis test was used for group comparisons. P-values were corrected for multiple testing by Benjamini-Hochberg.

A total of 61 food groups are presented, whereby the main food groups are highlighted in bold. The sum does not always add up since not all possible foods of the group are shown. Sum of cereals and of precooked meals, rather than total amounts of each, are indicated to distinguish their subgroups.

OMN: omnivore; VGN: vegan; OVL: ovo-lacto-vegetarian; PCV: pesco-vegetarian. PB: plant-based; ALT: alternative; ALC: alcohol

**Table S7.** Post-hoc analysis of the dietary intake of the main food groups by type of diet in the OMIVECA study.

|  | **OMN vs PCV** | **OMN vs OVL** | **OMN vs VGN** | **PCV vs OVL** | **PCV vs VGN** | **OVL vs VGN** |
| --- | --- | --- | --- | --- | --- | --- |
| Milk | <0.001 | <0.001 | <0.001 | 0.837 | <0.001 | <0.001 |
| Other dairy | 0.230 | 0.006 | <0.001 | 0.548 | <0.001 | <0.001 |
| Dairy desserts | 0.114 | 0.297 | <0.001 | 0.331 | <0.001 | <0.001 |
| **Total dairy** | <0.001 | <0.001 | <0.001 | 0.667 | <0.001 | <0.001 |
| **Egg** | 0.877 | 0.647 | <0.001 | 0.710 | <0.001 | <0.001 |
| Red meat | <0.001 | <0.001 | <0.001 | 0.004 | 0.001 | 0.210 |
| White meat | <0.001 | <0.001 | <0.001 | <0.001 | <0.001 | 0.123 |
| Processed meat | <0.001 | <0.001 | <0.001 | <0.001 | <0.001 | 0.123 |
| **Total meat** | <0.001 | <0.001 | <0.001 | <0.001 | <0.001 | 0.074 |
| White fish | 0.778 | <0.001 | <0.001 | <0.001 | <0.001 | 0.148 |
| Blue fish | 0.848 | <0.001 | <0.001 | <0.001 | <0.001 | 0.119 |
| Canned fish | 0.591 | <0.001 | <0.001 | <0.001 | <0.001 | 0.054 |
| Seafood | 0.482 | <0.001 | <0.001 | <0.001 | <0.001 | 0.698 |
| **Total fish & seafood** | 0.699 | <0.001 | <0.001 | <0.001 | <0.001 | 0.290 |
| **Potatoes** | 0.331 | 0.445 | 0.250 | 0.794 | 0.906 | 0.627 |
| Vegetables | 0.006 | <0.001 | <0.001 | 0.918 | 0.024 | 0.006 |
| Mushrooms | 0.010 | <0.001 | <0.001 | 0.618 | 0.618 | 0.951 |
| **Vegetables & mushrooms** | 0.003 | <0.001 | <0.001 | 0.912 | 0.046 | 0.009 |
| **Legumes** | <0.001 | <0.001 | <0.001 | 0.506 | 0.003 | 0.002 |
| **Fruits** | 0.283 | 0.283 | 0.980 | 0.980 | 0.283 | 0.283 |
| **Nuts** | 0.100 | 0.005 | <0.001 | 0.924 | <0.001 | <0.001 |
| Whole grain cereal | 0.002 | 0.004 | 0.002 | 0.285 | 0.497 | 0.641 |
| Refined cereal | 0.043 | 0.005 | <0.001 | 0.828 | 0.288 | 0.141 |
| **Sum of cereals** | 0.445 | 0.758 | 0.413 | 0.445 | 0.413 | 0.445 |
| Snacks | 0.735 | 0.478 | 0.486 | 0.478 | 0.871 | 0.478 |
| Pastries | 0.051 | 0.109 | <0.001 | 0.291 | 0.145 | <0.001 |
| Vegan pastries | <0.001 | <0.001 | <0.001 | 0.094 | <0.001 | 0.003 |
| **Total pastries** | 0.142 | 0.746 | 0.039 | 0.263 | 0.969 | 0.142 |
| **Sugars** | 0.144 | 0.018 | <0.001 | 0.895 | 0.009 | 0.001 |
| Dark chocolate | 0.159 | 0.159 | 0.584 | 0.584 | 0.365 | 0.468 |
| Olive oil | 0.978 | 0.157 | 0.978 | 0.304 | 0.978 | 0.259 |
| Vegetable fat | 0.925 | 0.181 | 0.668 | 0.367 | 0.709 | 0.373 |
| Animal fat | 0.947 | 0.253 | <0.001 | 0.505 | <0.001 | <0.001 |
| **Coffee & tea** | 0.021 | 0.006 | 0.175 | 0.890 | 0.413 | 0.413 |
| Sugary drinks | 0.756 | 0.261 | 0.317 | 0.756 | 0.756 | 0.802 |
| Sweetened drinks | 0.380 | 0.188 | 0.188 | 0.951 | 0.951 | 0.951 |
| Bottled juices | 0.254 | 0.254 | 0.254 | 0.896 | 0.896 | 0.896 |
| **Total non-alc. beverages** | 0.120 | 0.007 | 0.011 | 0.867 | 0.867 | 0.867 |
| **PB beverages** | <0.001 | <0.001 | <0.001 | 0.761 | 0.002 | <0.001 |
| Wine | 0.237 | 0.677 | 0.237 | 0.341 | 0.173 | 0.237 |
| Beer | 0.459 | 0.288 | 0.288 | 0.774 | 0.288 | 0.288 |
| Spirits | 0.103 | 0.022 | 0.004 | 0.897 | 0.594 | 0.483 |
| Non-alcoholic beer | 0.120 | 0.007 | 0.011 | 0.867 | 0.867 | 0.867 |
| **Total alc. beverages** | 0.369 | 0.282 | 0.084 | 0.905 | 0.084 | 0.084 |
| Spices | 0.030 | <0.001 | <0.001 | 0.254 | 0.016 | 0.022 |
| Salt | 0.410 | 0.614 | 0.410 | 0.646 | 0.646 | 0.656 |
| Omnivorous precooked meals | <0.001 | <0.001 | <0.001 | NA | NA | <0.001 |
| Vegan precooked meals | <0.001 | <0.001 | <0.001 | 0.015 | <0.001 | <0.001 |
| **Sum of precooked meals** | 0.259 | 0.247 | <0.001 | 0.725 | 0.025 | <0.001 |
| Sauces | 0.364 | 0.012 | 0.003 | 0.934 | 0.157 | 0.099 |
| Soups & creams | 0.307 | 0.002 | 0.001 | 0.307 | 0.102 | 0.248 |
| **Prepared meals** | 0.849 | <0.001 | <0.001 | 0.062 | 0.001 | 0.759 |
| Meat alternatives | <0.001 | <0.001 | <0.001 | 0.182 | 0.182 | 0.688 |
| **PB alt. protein** | <0.001 | <0.001 | <0.001 | 0.013 | <0.001 | <0.001 |
| Seaweed | 0.071 | <0.001 | <0.001 | 0.657 | 0.224 | 0.224 |
| **Spreads** | 0.010 | <0.001 | <0.001 | 0.044 | 0.039 | 0.899 |
| Kombucha | 0.204 | 0.001 | 0.001 | 0.593 | 0.593 | 0.830 |
| Smoothies | 0.294 | 0.108 | 0.180 | 0.108 | 0.134 | 0.819 |
| **Vegan dairy-like foods** | <0.001 | <0.001 | <0.001 | 0.505 | <0.001 | 0.001 |

Kruskal-Wallis test was used for group comparisons. P-values were corrected for multiple testing by Benjamini-Hochberg

OMN: omnivore/omnivorous; VGN: vegan; OVL: ovo-lacto-vegetarian; PCV: pesco-vegetarian. PB: plant-based; alt: alternative; alc: alcohol.

**Table S8.** Raw dietary intake of the main food groups by type of diet in the OMIVECA study.

|  | **All**  **n=760** | **OMN**  ***n=599*** | **PCV**  ***n=31*** | **OVL**  ***n=73*** | **VGN**  ***n=57*** | **p-value** |
| --- | --- | --- | --- | --- | --- | --- |
| Milk | 201.4 (214.3) | 237.5 (217.6) | 131.4 (191.8) | 91.8 (134.6) | 0.0 (0.0) | <0.001 |
| Other dairy | 108.2 (95.5) | 120.0 (93.5) | 94.6 (62.5) | 101.5 (107.5) | 0.4 (2.1) | <0.001 |
| Dairy desserts | 6.5 (12.9) | 7.5 (14.0) | 4.1 (6.8) | 4.7 (6.8) | 0.3 (1.1) | <0.001 |
| **Total dairy** | 316.2 (254.1) | 365.1 (246.1) | 230.0 (236.9) | 198.0 (195.4) | 0.6 (2.4) | <0.001 |
| **Egg** | 40.8 (41.2) | 44.1 (41.6) | 37.1 (16.6) | 47.8 (43.8) | 0.2 (0.8) | <0.001 |
| Red meat | 37.5 (40.9) | 47.4 (40.8) | 4.2 (11.0) | 0.2 (1.4) | 0.0 (0.0) | <0.001 |
| White meat | 56.3 (60.8) | 71.0 (60.4) | 6.5 (11.9) | 0.4 (1.7) | 0.0 (0.0) | <0.001 |
| Processed meat | 28.8 (33.8) | 36.2 (34.3) | 4.7 (14.3) | 0.7 (4.9) | 0.0 (0.0) | <0.001 |
| **Total meat** | 124.9 (107.4) | 157.5 (97.7) | 16.1 (33.9) | 1.3 (7.3) | 0.0 (0.0) | <0.001 |
| White fish | 20.7 (21.3) | 24.9 (21.0) | 25.6 (21.8) | 0.3 (1.5) | 0.0 (0.0) | <0.001 |
| Blue fish | 32.1 (28.0) | 38.3 (26.3) | 40.4 (29.5) | 1.8 (7.4) | 0.2 (1.2) | <0.001 |
| Canned fish | 10.6 (11.5) | 12.7 (11.5) | 13.7 (11.1) | 0.5 (2.0) | 0.0 (0.0) | <0.001 |
| Seafood | 19.4 (22.2) | 22.7 (20.3) | 36.3 (45.6) | 0.5 (2.8) | 0.1 (0.7) | <0.001 |
| **Total fish & seafood** | 72.2 (54.3) | 85.9 (45.6) | 102.3 (81.3) | 2.6 (9.3) | 0.3 (1.4) | <0.001 |
| **Potatoes** | 54.4 (41.5) | 56.3 (42.5) | 44.0 (29.4) | 49.8 (37.7) | 46.5 (39.7) | 0.105 |
| Vegetables | 423.8 (279.7) | 389.1 (260.4) | 500.4 (200.5) | 491.7 (239.8) | 660.6 (401.6) | <0.001 |
| Mushrooms | 31.7 (41.8) | 26.3 (35.4) | 47.4 (47.2) | 57.2 (70.7) | 46.7 (34.8) | <0.001 |
| **Vegetables & mushrooms** | 455.5 (294.1) | 415.4 (270.2) | 547.8 (218.8) | 548.9 (287.5) | 707.3 (404.2) | <0.001 |
| **Legumes** | 98.4 (74.1) | 84.3 (63.3) | 124.3 (62.3) | 133.5 (70.7) | 186.7 (105.6) | <0.001 |
| **Fruits** | 472.3 (344.3) | 467.4 (355.5) | 501.8 (240.8) | 490.5 (291.5) | 484.1 (339.0) | 0.200 |
| **Nuts** | 24.3 (25.9) | 21.0 (21.5) | 27.2 (21.7) | 27.5 (23.2) | 53.6 (46.7) | <0.001 |
| Whole grain cereal | 52.7 (54.8) | 48.4 (54.1) | 76.4 (53.2) | 64.3 (55.3) | 69.8 (54.4) | <0.001 |
| Refined cereal | 85.1 (72.2) | 91.0 (75.9) | 67.7 (56.2) | 65.9 (51.0) | 56.9 (48.1) | <0.001 |
| **Sum of cereals** | 137.7 (82.8) | 139.4 (86.9) | 144.2 (61.6) | 130.2 (62.1) | 126.7 (71.5) | 0.472 |
| Snacks | 6.8 (10.7) | 6.9 (11.3) | 6.3 (8.8) | 6.7 (6.1) | 6.5 (9.6) | 0.371 |
| Pastries | 27.6 (37.2) | 30.1 (39.9) | 22.0 (29.1) | 22.6 (23.4) | 10.2 (12.5) | <0.001 |
| Vegan pastries | 1.5 (6.7) | 0.4 (5.0) | 2.4 (5.9) | 4.6 (10.8) | 8.1 (10.4) | <0.001 |
| **Total pastries** | 29.1 (38.0) | 30.6 (40.3) | 24.3 (31.4) | 27.2 (30.6) | 18.2 (18.4) | 0.026 |
| **Sugars** | 11.0 (13.4) | 11.9 (13.7) | 9.8 (14.8) | 8.8 (11.7) | 5.1 (8.2) | <0.001 |
| Dark chocolate | 5.3 (8.3) | 4.9 (7.9) | 8.7 (12.9) | 6.2 (7.2) | 6.2 (10.5) | 0.073 |
| Olive oil | 24.3 (17.6) | 23.8 (17.1) | 23.9 (15.1) | 28.7 (20.1) | 24.8 (20.6) | 0.099 |
| Vegetable fat | 26.0 (18.4) | 25.4 (17.5) | 25.0 (14.8) | 30.2 (20.2) | 28.6 (25.0) | 0.087 |
| Animal fat | 1.0 (2.9) | 1.1 (3.2) | 0.8 (1.7) | 0.6 (1.1) | 0.0 (0.0) | <0.001 |
| **Coffee & tea** | 69.5 (64.8) | 64.2 (61.9) | 101.9 (89.5) | 88.1 (61.6) | 83.6 (72.6) | <0.001 |
| Sugary drinks | 16.3 (41.8) | 18.0 (45.5) | 14.2 (31.6) | 8.1 (16.1) | 9.6 (22.0) | 0.108 |
| Sweetened drinks | 18.4 (45.6) | 20.6 (49.5) | 8.2 (16.8) | 11.3 (23.8) | 9.9 (28.8) | 0.073 |
| Bottled juices | 8.3 (26.3) | 9.3 (28.3) | 1.5 (4.0) | 4.0 (12.2) | 6.5 (24.3) | 0.099 |
| **Total non-alc. beverages** | 42.9 (73.1) | 47.9 (79.1) | 23.9 (34.4) | 23.4 (34.7) | 26.1 (45.8) | <0.001 |
| **PB beverages** | 67.2 (133.6) | 32.7 (83.8) | 159.4 (173.1) | 147.2 (167.7) | 277.6 (209.7) | <0.001 |
| Wine | 8.4 (21.9) | 8.2 (22.1) | 14.2 (26.5) | 9.6 (22.1) | 6.4 (15.0) | 0.137 |
| Beer | 38.5 (72.8) | 37.2 (73.7) | 34.7 (46.0) | 56.5 (83.4) | 31.4 (57.8) | 0.174 |
| Spirits | 2.2 (4.8) | 2.5 (5.2) | 1.1 (2.5) | 1.2 (3.1) | 0.8 (2.0) | <0.001 |
| Non-alcoholic beer | 7.0 (26.7) | 5.4 (24.2) | 21.9 (43.5) | 12.5 (33.6) | 8.2 (27.0) | <0.001 |
| **Total alc. beverages** | 49.1 (83.1) | 47.8 (84.7) | 50.1 (63.0) | 67.3 (88.3) | 38.6 (65.3) | 0.062 |
| Spices | 5.6 (6.0) | 4.8 (5.2) | 6.5 (5.0) | 7.5 (5.6) | 11.4 (9.9) | <0.001 |
| Salt | 3.6 (2.8) | 3.5 (2.7) | 4.6 (3.4) | 3.9 (3.2) | 3.8 (2.4) | 0.143 |
| Omnivorous precooked meals | 27.6 (31.5) | 35.0 (31.6) | 0.0 (0.0) | 0.0 (0.0) | 0.0 (0.0) | <0.001 |
| Vegan precooked meals | 7.2 (17.6) | 1.6 (5.4) | 34.6 (34.4) | 32.3 (30.1) | 19.2 (18.6) | <0.001 |
| **Sum of precooked meals** | 34.8 (31.8) | 36.6 (32.5) | 34.6 (34.4) | 32.3 (30.1) | 19.2 (18.6) | <0.001 |
| Sauces | 13.5 (14.6) | 14.1 (14.9) | 10.4 (13.5) | 12.6 (13.2) | 10.0 (12.4) | 0.010 |
| Soups & creams | 10.3 (20.1) | 11.0 (20.1) | 7.9 (16.8) | 11.0 (24.6) | 4.1 (12.5) | 0.002 |
| **Prepared meals** | 60.3 (52.9) | 61.1 (51.9) | 64.6 (57.0) | 66.5 (59.9) | 42.4 (49.6) | 0.001 |
| Meat alternatives | 13.6 (34.1) | 3.2 (9.7) | 44.3 (52.7) | 49.6 (52.8) | 59.0 (65.0) | <0.001 |
| **PB alt. protein** | 36.8 (83.0) | 7.0 (16.4) | 91.5 (92.9) | 123.5 (92.2) | 208.7 (154.8) | <0.001 |
| Seaweed | 0.2 (0.4) | 0.1 (0.3) | 0.4 (0.8) | 0.3 (0.6) | 0.4 (0.5) | <0.001 |
| **Spreads** | 7.3 (10.0) | 5.8 (8.2) | 9.5 (10.5) | 13.6 (11.7) | 14.8 (16.8) | <0.001 |
| Kombucha | 2.8 (13.5) | 2.1 (11.3) | 2.7 (6.7) | 3.5 (6.9) | 9.5 (30.8) | <0.001 |
| Smoothies | 6.8 (19.3) | 5.9 (16.7) | 2.7 (6.7) | 11.5 (31.2) | 12.7 (26.3) | 0.050 |
| **Vegan dairy-like foods** | 18.1 (41.5) | 11.1 (30.9) | 31.5 (52.4) | 37.5 (59.1) | 60.0 (65.3) | <0.001 |

Raw dietary intakes are presented as means and standard deviations (SD) to facilitate interpretation. As the variables did not follow a normal distribution, the Kruskal-Wallis test was used for group comparisons. P-values were corrected for multiple testing by Benjamini-Hochberg.

A total of 61 food groups are presented, whereby the main food groups are highlighted in bold. The sum does not always add up since not all possible foods of the group are shown. Sum of cereals and of precooked meals, rather than total amounts of each, are indicated to distinguish their subgroups.

OMN: omnivore/omnivorous; VGN: vegan; OVL: ovo-lacto-vegetarian; PCV: pesco-vegetarian; PB: plant-based; alt: alternative; alc: alcohol.

**Table S9.** Logistic regression model: Adherence to high hPDI by diet, adjusted.

|  |  | **Model 1** | | **Model 2** | |
| --- | --- | --- | --- | --- | --- |
|  |  | OR | (95% CI) | OR | (95% CI) |
| Diet |  |  |  |  |  |
|  | Omnivorous (Ref) | 1.00 | - | 1.00 | - |
|  | Vegan | 37.14 | (16.22 – 97.61) | 42.26 | (17.81 – 115.31) |
|  | Ovo-lacto-vegetarian | 7.89 | (4.58 – 13.74) | 8.02 | (4.62 – 14.09) |
|  | Pesco-vegetarian | 4.33 | (1.97 – 9.40) | 3.95 | (1.77 – 8.72) |

Healthy Provegetarian Diet Index (hPDI) ranges from 18 to 90 points. It is categorized in “High” for 65 points or more and “Low” for less than 65 points. OR: Odds Ratio; CI: Confidential interval; Ref: Reference.

Model 1: Adjusted for sex, age in years, participation center (Granada, others) and profession (nutrition, health sciences, other).

Model 2: Model 1 + BMI, physical activity and total energy.

**Table S10:** Factor loadings of each variable in PCA in the OMIVECA study.

| **Food groups** | **Component 1**  **(15.0%)** | **Component 2**  **(7.2%)** | **Component 3**  **(5.5%)** |
| --- | --- | --- | --- |
| Total dairy | **-0.547** | 0.024 | -0.048 |
| Egg | **-0.343** | -0.159 | 0.194 |
| Total meat | **-0.676** | **0.209** | -0.140 |
| Total fish & seafood | **-0.556** | -0.082 | 0.032 |
| Potatoes | -0.059 | 0.106 | **-0.216** |
| Vegetables & mushrooms | **0.255** | **-0.484** | **0.229** |
| Legumes | **0.426** | **-0.304** | -0.025 |
| Fruits | -0.029 | **-0.467** | -0.113 |
| Nuts | **0.274** | **-0.453** | -0.086 |
| Whole grain cereal | 0.035 | **-0.516** | 0.052 |
| Refined cereal | -0.176 | **0.287** | -0.139 |
| Pastries | -0.031 | **0.550** | **-0.255** |
| Snacks | 0.156 | **0.592** | -0.028 |
| Vegetable fat | 0.017 | 0.127 | **0.553** |
| Animal fat | -0.118 | 0.164 | 0.118 |
| Sugars | -0.071 | **0.468** | 0.009 |
| Coffee & tea | 0.045 | -0.176 | **0.556** |
| Total non-alc. beverages | -0.092 | **0.451** | **0.247** |
| Total alc. beverages | 0.024 | **0.209** | **0.406** |
| Vegan precooked meals | **0.644** | 0.042 | 0.039 |
| Omnivorous precooked meals | **-0.389** | **0.496** | **-0.202** |
| Sauces | -0.005 | **0.482** | -0.155 |
| Soups & creams | 0.013 | **0.216** | **-0.301** |
| Spices | **0.257** | **-0.200** | **0.304** |
| Salt | -0.029 | 0.069 | **0.672** |
| PB alt. protein | **0.783** | -0.165 | 0.059 |
| Seaweed | **0.235** | -0.061 | **0.226** |
| Vegan pastries | **0.440** | 0.041 | -0.038 |
| Spreads | **0.409** | -0.127 | 0.166 |
| PB beverages | **0.555** | -0.191 | **0.233** |
| Smoothies | 0.197 | -0.048 | -0.063 |
| Vegan dairy-like foods | **0.455** | -0.110 | 0.144 |

In parentheses the percentage of the total explained variance is presented.

Factor loading in bold when absolute value was higher than 0.2.

OMN: omnivorous; VGN: vegan; PB: plant-based; ALT: alternative; ALC: alcohol.

**Table S11:** Description of food groups by PCA clusters in the OMIVECA study.

|  |  | **Cluster 1: Mixed OMN diet n=323** | **Cluster 2: PBD n=153** | **Cluster 3: Unhealthy OMN diet n=284** | **p-value** |
| --- | --- | --- | --- | --- | --- |
| Diet ^a^ | |  |  |  | <0.001 |
|  | Omnivorous | 307 (95.0%) | 10 (6.5%) | 282 (99.3%) |  |
|  | Vegan | 0 (0.0%) | 57 (37.3%) | 0 (0.0%) |  |
|  | Ovo-lacto-vegetarian | 5 (1.5%) | 67 (43.8%) | 1 (0.4%) |  |
|  | Pesco-vegetarian | 11 (3.5%) | 19 (12.4%) | 1 (0.4%) |  |
| Age ^b^ |  | 22.0 (20.0;29.0) | 28.0 (23.0;36.0) | 20.0 (19.0;24.0) | <0.001 |
| hPDI categorized ^a^ | |  |  |  | <0.001 |
|  | Low (<50p) | 31 (9.6%) | 7 (4.6%) | 176 (62.0%) |  |
|  | Intermediate (51-64p) | 214 (66.3%) | 40 (26.1%) | 107 (37.7%) |  |
|  | High (≥65p) | 78 (24.1%) | 106 (69.3%) | 1 (0.4%) |  |
| Sex ^c^ | Female | 246 (76.2%) | 120 (78.4%) | 190 (66.9%) | 0.011 |
| BMI ^b^ |  | 22.3 (20.6;24.1) | 21.8 (20.1;23.9) | 22.5 (20.4;25.1) | 0.196 |
| Total dairy ^d^ | | 145.2 (94.4) | 39.1 (57.0) | 142.9 (84.1) | <0.001 |
| Egg ^d^ | | 21.5 (20.7) | 10.6 (13.0) | 13.9 (10.2) | <0.001 |
| Total meat ^d^ | | 54.7 (32.7) | 2.9 (12.9) | 66.0 (29.1) | <0.001 |
| Total fish & seafood ^d^ | | 36.5 (20.2) | 7.1 (20.9) | 31.9 (16.4) | <0.001 |
| Potatoes ^d^ | | 20.1 (15.7) | 18.2 (12.7) | 23.9 (16.0) | <0.001 |
| Vegetables & mushrooms ^d^ | | 204.5 (100.1) | 236.8 (101.7) | 117.8 (68.6) | <0.001 |
| Legumes ^d^ | | 37.1 (23.8) | 57.8 (26.1) | 28.1 (19.3) | <0.001 |
| Fruits ^d^ | | 215.8 (132.1) | 189.1 (116.7) | 139.1 (77.6) | <0.001 |
| Nuts ^d^ | | 10.6 (9.0) | 13.9 (10.5) | 5.1 (5.0) | <0.001 |
| Whole grain cereal ^d^ | | 27.5 (22.5) | 24.6 (17.2) | 10.1 (12.3) | <0.001 |
| Refined cereal ^d^ | | 28.6 (22.0) | 23.7 (16.8) | 40.7 (23.7) | <0.001 |
| Pastries ^d^ | | 6.6 (6.0) | 6.3 (7.1) | 16.8 (14.5) | <0.001 |
| Snacks ^d^ | | 1.4 (1.7) | 2.5 (2.9) | 4.1 (4.4) | <0.001 |
| Vegetable fat ^d^ | | 10.1 (6.0) | 11.1 (6.8) | 9.7 (6.5) | 0.033 |
| Animal fat ^d^ | | 0.3 (1.1) | 0.2 (0.4) | 0.6 (1.4) | <0.001 |
| Sugars ^d^ | | 3.1 (3.6) | 2.7 (3.8) | 6.3 (5.5) | <0.001 |
| Coffee & tea ^d^ | | 33.9 (30.6) | 35.4 (31.4) | 19.9 (21.8) | <0.001 |
| Total non-alc. beverages ^d^ | | 10.4 (15.9) | 9.5 (15.0) | 28.8 (43.1) | <0.001 |
| Total alc. beverages ^d^ | | 16.9 (25.7) | 24.4 (38.6) | 21.5 (41.4) | 0.736 |
| Vegan precooked meals ^d^ | | 0.8 (2.4) | 10.2 (9.4) | 0.9 (3.1) | <0.001 |
| Omnivorous precooked meals ^d^ | | 8.3 (6.4) | 0.4 (1.8) | 19.2 (13.0) | <0.001 |
| Sauces ^d^ | | 3.4 (3.4) | 4.3 (4.1) | 7.7 (5.9) | <0.001 |
| Soups & creams ^d^ | | 2.5 (5.2) | 2.7 (6.8) | 6.1 (9.0) | <0.001 |
| Spices ^d^ | | 2.3 (2.2) | 3.4 (2.6) | 1.4 (1.5) | <0.001 |
| Salt ^d^ | | 1.5 (1.2) | 1.6 (1.2) | 1.2 (1.0) | 0.001 |
| PB alt. protein ^d^ | | 4.6 (9.5) | 56.2 (39.8) | 1.8 (5.3) | <0.001 |
| Seaweed ^d^ | | 0.1 (0.1) | 0.1 (0.3) | <0.1 (0.1) | <0.001 |
| Vegan pastries ^d^ | | 0.1 (0.3) | 2.5 (4.7) | 0.1 (0.7) | <0.001 |
| Spreads ^d^ | | 2.6 (3.1) | 5.1 (4.0) | 1.8 (2.9) | <0.001 |
| PB beverages ^d^ | | 17.9 (39.3) | 85.3 (82.0) | 5.9 (21.6) | <0.001 |
| Smoothies ^d^ | | 2.3 (7.1) | 4.4 (10.8) | 2.3 (6.1) | 0.039 |
| Vegan dairy-like foods ^d^ | | 4.7 (12.0) | 20.9 (27.4) | 3.0 (9.0) | <0.001 |

^a^ Categorical variables are expressed as n (%) and analyzed using the Fisher’s exact test, ^c^ Pearson chi-square test.

^b^ Age and BMI are presented as median and intervale range and compared using the Kruskal-Wallis test and corrected for multiple testing by Benjamini-Hochberg.

^d^ Dietary intakes (adjusted per 1,000 kcal/day) are presented as means and standard deviations and they were compared using the Kruskal-Wallis test and corrected for multiple testing by Benjamini-Hochberg.

The food groups included were the 32 used to explore PCA. OMN: omnivorous; PBD: Plant-based diet; PB: plant-based; ALT: alternative; ALC: alcohol.

**Table S12:** Factor loadings and VIP of the three first components in PLS-DA in the OMIVECA study.

|  | Factor loadings | | | VIP | | |
| --- | --- | --- | --- | --- | --- | --- |
|  | Comp. 1 | Comp. 2 | Comp. 3 | Comp. 1 | Comp. 2 | Comp. 3 |
| Total dairy | **-0.225** | **0.167** | **0.042** | **1.275** | **1.225** | **1.170** |
| Egg | -0.093 | 0.318 | 0.194 | 0.525 | 0.880 | 0.902 |
| Total meat | **-0.362** | **-0.233** | **-0.086** | **2.049** | **1.946** | **1.860** |
| Total fish & seafood | **-0.282** | -0.135 | 0.477 | **1.593** | **1.486** | **1.635** |
| Potatoes | -0.049 | 0.037 | -0.067 | 0.276 | 0.265 | 0.278 |
| Vegetables & mushrooms | 0.152 | -0.223 | 0.213 | 0.857 | 0.937 | 0.965 |
| Legumes | **0.220** | **-0.128** | **0.000** | **1.242** | **1.171** | **1.116** |
| Fruits | 0.015 | -0.029 | 0.247 | 0.084 | 0.102 | 0.434 |
| Nuts | 0.153 | **-0.326** | **-0.009** | 0.865 | **1.093** | **1.042** |
| Whole grain cereal | 0.075 | -0.029 | 0.272 | 0.422 | 0.391 | 0.597 |
| Refined cereal | -0.105 | 0.067 | -0.141 | 0.593 | 0.562 | 0.588 |
| Pastries | -0.091 | 0.189 | -0.181 | 0.516 | 0.642 | 0.686 |
| Snacks | -0.005 | 0.102 | -0.228 | 0.029 | 0.238 | 0.452 |
| Vegetable fat | 0.036 | 0.016 | -0.030 | 0.205 | 0.191 | 0.189 |
| Animal fat | -0.058 | 0.055 | -0.028 | 0.328 | 0.325 | 0.314 |
| Sugars | -0.089 | 0.113 | -0.119 | 0.506 | 0.531 | 0.545 |
| Coffee & tea | 0.065 | 0.029 | 0.246 | 0.370 | 0.344 | 0.535 |
| Total non-alc. beverages | -0.071 | 0.018 | -0.168 | 0.399 | 0.367 | 0.453 |
| Total alc. beverages | 0.012 | 0.104 | 0.000 | 0.065 | 0.249 | 0.237 |
| Vegan precooked meals | **0.344** | **0.621** | **0.174** | **1.944** | **2.285** | **2.198** |
| Omnivorous precooked meals | **-0.264** | **-0.115** | **-0.230** | **1.495** | **1.390** | **1.382** |
| Sauces | -0.062 | 0.125 | -0.208 | 0.350 | 0.431 | 0.544 |
| Soups & creams | -0.040 | 0.123 | -0.109 | 0.226 | 0.352 | 0.384 |
| Spices | 0.152 | -0.187 | -0.017 | 0.860 | 0.896 | 0.855 |
| Salt | 0.039 | 0.026 | 0.095 | 0.220 | 0.209 | 0.257 |
| PB alt. protein | **0.428** | **-0.003** | **-0.311** | **2.422** | **2.209** | **2.171** |
| Seaweed | 0.113 | -0.014 | 0.105 | 0.638 | 0.583 | 0.584 |
| Vegan pastries | **0.195** | **-0.116** | **-0.251** | **1.104** | **1.042** | **1.083** |
| Spreads | 0.168 | -0.021 | 0.081 | 0.952 | 0.870 | 0.841 |
| PB beverages | **0.269** | **-0.176** | **0.027** | **1.524** | **1.449** | **1.381** |
| Smoothies | 0.049 | -0.107 | -0.027 | 0.279 | 0.355 | 0.341 |
| Vegan dairy-like foods | 0.175 | -0.148 | 0.071 | 0.989 | 0.965 | 0.928 |

In bold for the values with a Variable Importance in Projection (VIP) higher than 1.0.

**Table S13:** Post-hoc analysis of omnivores classified in the three HCA clusters in the OMIVECA study.

|  |  | **PBD vs UNHEALTHY** | **PBD vs**  **MIXED** | **UNHEALTHY vs MIXED** |
| --- | --- | --- | --- | --- |
| Age |  | 0.469 | 0.667 | 0.469 |
| hPDI categorized | | <0.001 | <0.001 | <0.001 |
| Sex |  | 0.027 | 0.099 | 0.099 |
| BMI |  | 0.201 | 0.201 | 0.366 |
| Milk |  | <0.001 | <0.001 | 0.825 |
| Total dairy | | 0.001 | <0.001 | 0.280 |
| Egg | | 0.005 | 0.991 | <0.001 |
| Red meat | | <0.001 | 0.011 | 0.001 |
| White meat | | 0.694 | 0.868 | 0.447 |
| Processed meat | | <0.001 | 0.001 | 0.017 |
| Total meat | | 0.008 | 0.028 | 0.158 |
| Total fish & seafood | | 0.390 | 0.862 | 0.193 |
| Potatoes | | 0.002 | 0.096 | 0.003 |
| Vegetables | | <0.001 | 0.034 | <0.001 |
| Vegetables & mushrooms | | <0.001 | 0.017 | <0.001 |
| Legumes | | 0.005 | 0.869 | <0.001 |
| Fruits | | <0.001 | 0.472 | <0.001 |
| Nuts | | <0.001 | 0.002 | <0.001 |
| Whole grain cereal | | <0.001 | 0.041 | <0.001 |
| Refined cereal | | <0.001 | 0.001 | 0.316 |
| Pastries | | <0.001 | 0.001 | <0.001 |
| Snacks | | <0.001 | 0.178 | <0.001 |
| Vegetable fat | | 0.675 | 0.562 | 0.127 |
| Animal fat | | 0.023 | 0.135 | 0.026 |
| Sugars | | <0.001 | 0.062 | <0.001 |
| Coffee & tea | | 0.001 | 0.001 | 0.323 |
| Total non-alc. beverages | | <0.001 | 0.386 | <0.001 |
| Total alc. beverages | | 0.069 | 0.498 | 0.051 |
| Vegan precooked meals | | 0.157 | 0.190 | 0.384 |
| Omnivorous precooked meals | | <0.001 | 0.001 | <0.001 |
| Total precooked meals | | <0.001 | 0.007 | <0.001 |
| Sauces | | <0.001 | 0.043 | <0.001 |
| Soups & creams | | 0.001 | 0.024 | 0.007 |
| Spices | | <0.001 | <0.001 | 0.003 |
| Salt | | 0.039 | 0.039 | 0.326 |
| PB alt. protein | | <0.001 | <0.001 | 0.017 |
| Seaweed | | 0.006 | 0.005 | 0.785 |
| Vegan pastries | | 0.659 | 0.540 | 0.540 |
| Spreads | | 0.154 | 0.224 | 0.273 |
| PB beverages | | <0.001 | <0.001 | 0.043 |
| Smoothies | | 0.017 | 0.001 | 0.337 |
| Vegan dairy-like foods | | <0.001 | <0.001 | 0.262 |

Kruskal-Wallis test was used for group comparisons. P-values were corrected for multiple testing by Benjamini-Hochberg.

OMN: omnivorous; PBD: Plant-based diet; alt: alternative; alc: alcohol.

**Table S14:** Factor loadings derived from PCA of the four main sensitivity analyses in the OMIVECA study.

|  | **Dimension 1** | | | | **Dimension 2** | | | | **Dimension 3** | | | |
| --- | --- | --- | --- | --- | --- | --- | --- | --- | --- | --- | --- | --- |
|  | **Female** | **Nutrition** | **>25y** | **OTN** | **Female** | **Nutrition** | **>25y** | **OTN** | **Female** | **Nutrition** | **>25y** | **OTN** |
| Variance explained (%) | 14.9 | 13.2 | 16.4 | 16.6 | 7.3 | 7.4 | 7.5 | 7.4 | 5.8 | 5.8 | 6.1 | 5.7 |
| Total dairy | -0.559 | -0.494 | -0.663 | -0.584 | - | - | - | - | - | - | - | - |
| Egg | -0.309 | -0.282 | -0.343 | -0.352 |  | -0.284 |  |  |  |  |  | 0.316 |
| Total meat | -0.657 | -0.655 | -0.722 | -0.704 | 0.255 | 0.221 |  | 0.226 | - | - | - | - |
| Total fish & seafood | -0.583 | -0.453 | -0.643 | -0.631 | - | - | - | - | - | - | - | - |
| Potatoes | - | - | - | - | - | - | 0.364 | 0.214 | -0.267 |  | -0.299 | -0.311 |
| Vegetables & mushrooms | - | 0.211 | 0.306 | 0.312 | -0.508 | -0.484 | -0.376 | -0.477 |  |  |  |  |
| Legumes | 0.377 | 0.321 | 0.536 | 0.496 | -0.324 |  | -0.219 | -0.343 | - | - | - | - |
| Fruits | - | - | - | - | -0.433 | -0.400 | -0.433 | -0.492 |  | -0.225 | -0.213 |  |
| Nuts | 0.241 | 0.306 | 0.292 | 0.243 | -0.469 | -0.395 | -0.455 | -0.482 |  | -0.207 |  |  |
| Whole grain cereal | - | - |  | - | -0.538 | -0.49 | -0.528 | -0.565 | - | - | - | - |
| Refined cereal |  | -0.231 |  |  | 0.320 | 0.215 | 0.514 | 0.442 | -0.287 |  | -0.2 |  |
| Pastries | - | - | - | - | 0.554 | 0.544 | 0.561 | 0.556 | -0.231 |  |  | -0.228 |
| Snacks |  | 0.209 |  |  | 0.574 | 0.617 | 0.422 | 0.532 |  |  | 0.244 |  |
| Vegetable fat | - | - | - | - | - | - | - | - | 0.457 | 0.504 | 0.516 | 0.658 |
| Animal fat |  |  | -0.239 |  |  | 0.283 |  |  |  |  |  |  |
| Sugars |  |  |  |  | 0.505 | 0.514 | 0.453 | 0.400 |  |  |  |  |
| Coffee & tea |  |  |  |  |  |  | -0.423 | -0.355 | 0.626 | 0.620 | 0.527 | 0.357 |
| Total non-alc. beverages |  |  | -0.224 |  | 0.423 | 0.409 | 0.268 | 0.376 | 0.349 | 0.317 | 0.528 |  |
| Total alc. beverages | - | - | - | - | - | - | - | - | 0.549 | 0.474 | 0.534 | 0.305 |
| Vegan precooked meals | 0.688 | 0.694 | 0.551 | 0.619 |  |  |  |  |  |  |  |  |
| Omnivorous precooked meals | -0.376 | -0.343 | -0.585 | -0.463 | 0.52 | 0.609 | 0.370 | 0.401 |  |  |  |  |
| Sauces |  |  |  |  | 0.538 | 0.443 | 0.538 | 0.561 |  |  |  |  |
| Soups & creams |  |  |  |  | 0.201 | 0.276 |  | 0.249 | -0.254 | -0.242 |  | -0.263 |
| Spices | 0.269 | 0.245 | 0.349 | 0.372 | -0.239 | -0.32 |  |  |  |  |  | 0.426 |
| Salt |  |  |  |  |  |  |  |  | 0.571 | 0.696 | 0.587 | 0.649 |
| PB alt. protein | 0.769 | 0.793 | 0.754 | 0.766 | -0.201 |  |  |  |  |  |  |  |
| Seaweed |  | 0.233 | 0.263 |  |  |  |  |  | 0.298 | 0.235 |  | 0.215 |
| Vegan pastries | 0.440 |  | 0.618 | 0.593 |  |  |  |  |  |  |  |  |
| Spreads | 0.379 | 0.352 | 0.448 | 0.464 |  |  |  |  |  |  | 0.25 |  |
| PB beverages | 0.514 | 0.551 | 0.541 | 0.536 | -0.233 |  |  | -0.254 | 0.274 |  | 0.218 |  |
| Smoothies |  |  | 0.228 | 0.246 |  |  |  |  |  |  |  |  |
| Vegan dairy-like foods | 0.456 | 0.449 | 0.409 | 0.423 |  |  |  |  |  |  |  |  |

Only presented the factor loadings higher than 0.2

>25y: older than 25 years old; OTN: other than nutrition group.

**Figure S1:** Adherence to high hPDI bivariate model plot by type of diet.


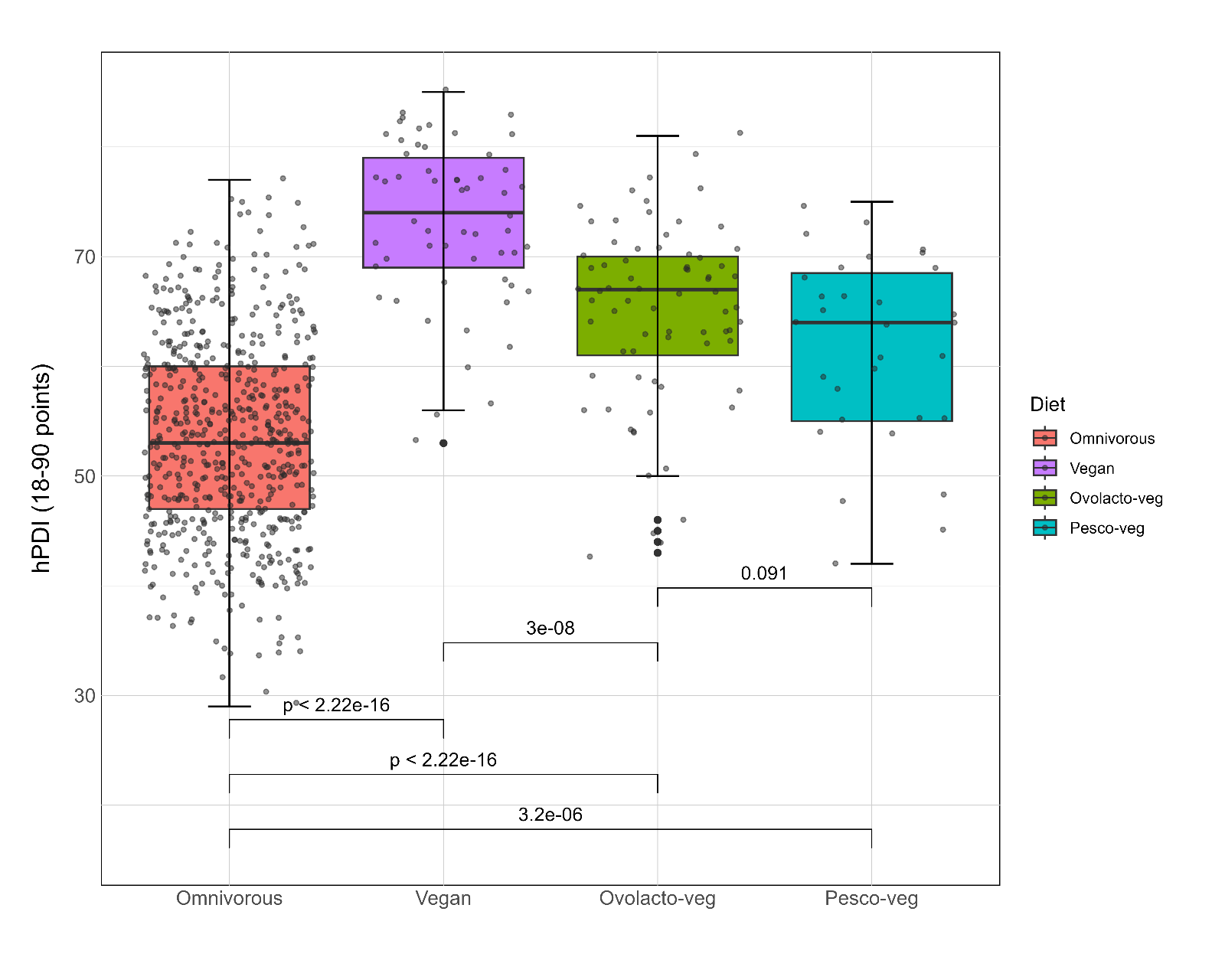


**Figure legend**: The boxplot shows the distribution of the healthy provegetarian diet index (hPDI) scores by dietary group. P-values were obtained from bivariate logistic regression analyses where hPDI was categorized into “High” (≥65p) and “Low” (>65p). Black dots represent individual hPDI scores as continuous values.
